# RANK rewires energy homeostasis in lung cancer cells and drives primary lung cancer

**DOI:** 10.1101/gad.304162.117

**Published:** 2017-10-15

**Authors:** Shuan Rao, Verena Sigl, Reiner Alois Wimmer, Maria Novatchkova, Alexander Jais, Gabriel Wagner, Stephan Handschuh, Iris Uribesalgo, Astrid Hagelkruys, Ivona Kozieradzki, Luigi Tortola, Roberto Nitsch, Shane J. Cronin, Michael Orthofer, Daniel Branstetter, Jude Canon, John Rossi, Manolo D'Arcangelo, Johan Botling, Patrick Micke, Linnea La Fleur, Karolina Edlund, Michael Bergqvist, Simon Ekman, Thomas Lendl, Helmut Popper, Hiroshi Takayanagi, Lukas Kenner, Fred R. Hirsch, William Dougall, Josef M. Penninger

**Affiliations:** 1 Institute of Molecular Biotechnology of the Austrian Academy of Sciences (IMBA), Vienna 1030, Austria;; 2 Department of Laboratory Medicine, Medical University Vienna, Vienna 1090, Austria;; 3 Department of Neuronal Control of Metabolism, Max Planck Institute for Metabolism Research, Cologne 50931, Germany;; 4 VetCore Facility for Research, University of Veterinary Medicine, Vienna 1220, Austria;; 5 Department of Pathology, Amgen, Inc., Seattle, Washington 98119, USA;; 6 Department of Oncology Research, Amgen, Inc., Seattle, Washington 98119, USA;; 7 Department of Molecular Sciences, Amgen, Inc., Seattle, Washington 98119, USA;; 8 University of Colorado Cancer Center, Aurora, Colorado 80045, USA;; 9 Department of Immunology, Genetics, and Pathology, Uppsala University, Uppsala 75185, Sweden;; 10 Leibniz Research Center for Working Environment and Human Factors, Dortmund 44139, Germany;; 11 Department of Oncology, Gavle Hospital, Gavle 80187, Sweden;; 12 Department of Oncology–Pathology, Karolinska Institutet, Stockholm 17177, Sweden;; 13 Gregor Mendel Institute of Molecular Plant Biology (GMI), Vienna 1030, Austria;; 14 Research Unit Molecular Lung and Pleura Pathology, Institute of Pathology, Medical University Graz, Graz 8036, Austria;; 15 Department of Immunology, Tokyo University, Tokyo 108-8639, Japan;; 16 Department of Clinical Pathology, Medical University Vienna, Vienna 1090, Austria;; 17 Ludwig Boltzmann Institute for Cancer Research, Vienna 1090, Austria;; 18 Unit of Pathology of Laboratory Animals, University of Veterinary Medicine Vienna, Vienna 1220, Austria

**Keywords:** RANK, energy homeostasis, lung cancer, lung cancer stem-like cells

## Abstract

Lung cancer is the leading cause of cancer deaths. Besides smoking, epidemiological studies have linked female sex hormones to lung cancer in women; however, the underlying mechanisms remain unclear. Here we report that the receptor activator of nuclear factor-kB (RANK), the key regulator of osteoclastogenesis, is frequently expressed in primary lung tumors, an active RANK pathway correlates with decreased survival, and pharmacologic RANK inhibition reduces tumor growth in patient-derived lung cancer xenografts. Clonal genetic inactivation of *KRas*^*G12D*^ in mouse lung epithelial cells markedly impairs the progression of *KRas*^*G12D*^-driven lung cancer, resulting in a significant survival advantage. Mechanistically, RANK rewires energy homeostasis in human and murine lung cancer cells and promotes expansion of lung cancer stem-like cells, which is blocked by inhibiting mitochondrial respiration. Our data also indicate survival differences in *KRas*^*G12D*^-driven lung cancer between male and female mice, and we show that female sex hormones can promote lung cancer progression via the RANK pathway. These data uncover a direct role for RANK in lung cancer and may explain why female sex hormones accelerate lung cancer development. Inhibition of RANK using the approved drug denosumab may be a therapeutic drug candidate for primary lung cancer.

## Abstract (precis)

Rao et al. report that RANK, the key regulator of osteoclastogenesis, is frequently expressed in primary lung tumors, and clonal genetic inactivation of RANK in mouse lung epithelial cells markedly impairs the progression of KRas^G12D^-driven lung cancer. RANK rewires energy homeostasis in human and murine lung cancer cells and promotes expansion of lung cancer stem-like cells.

The receptor activator of nuclear factor-κB (NF-κB) ligand (RANKL) is an essential cytokine that, upon binding to its cognate receptor, RANK, selectively activates intercellular signaling pathways to act as an essential regulator of osteoclastogenesis (Kong et al. 1999; Leibbrandt and Penninger 2008). In addition, RANKL/RANK have been found to control lymph node organogenesis (Kong et al. 1999), the development of intestinal M cells (Knoop et al. 2009), and AIRE^+^ thymic medullary epithelial cells (Rossi et al. 2007) as well as the formation of a lactating mammary gland in pregnancy (Fata et al. 2000). We and others have shown recently that RANKL/RANK are also involved in hormone- and *Brca1*-driven breast cancer in mice (Schramek et al. 2010; Nolan et al. 2016; Sigl et al. 2016).

RANKL inhibition with a monoclonal antibody called denosumab has been approved for osteoporosis and skeletal-related events in cancer (Branstetter et al. 2012; Brown-Glaberman and Stopeck 2013) and has been linked to delayed reoccurrence of breast tumors in an adjuvant setting (Gnant et al. 2015). During the clinical trials for bone metastasis, a post-hoc exploratory analysis surprisingly found that denosumab significantly improved survival of lung cancer patients with established metastasis (Scagliotti et al. 2012). Expression of estrogen and progesterone receptors also have been reported in lung cancer cells (Seattle et al. 1985; Cagle et al. 1990), and numerous studies have indicated more rapid progression of lung cancer in women (Belani et al. 2007; Remon et al. 2014), suggesting that sex hormones might affect the development of primary lung tumors. Since the RANKL/RANK system is known to be regulated by sex hormones (Fata et al. 2000; Beleut et al. 2010), we hypothesized that it might have a direct role in primary lung cancer.

## Results

### RANK is expressed in primary human lung cancer

**Figure 1.**
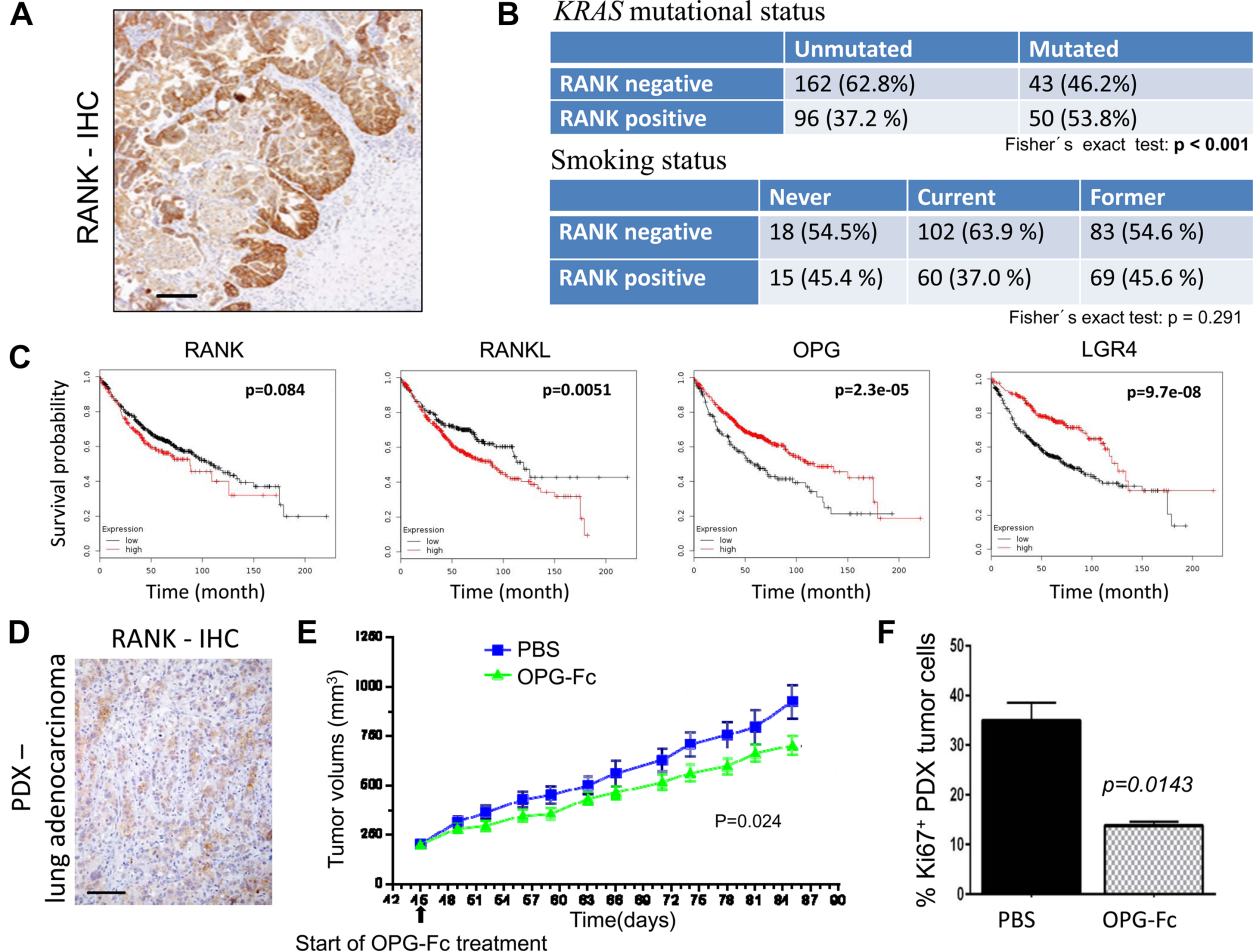
RANK is expressed in human lung tumors and controls growth of human lung cancer. (*A*) Representative RANK IHC in primary human NSCLC lung adenocarcinomas. Bars, 100 µm. (*B*) Cross-correlation matrixes to compare RANK protein expression (determined by IHC) in human lung tumors with *KRAS* mutational status and smoking. *n* = 364, ”Uppsala” cohort with early stage treatment-naïve resected lung cancer, including squamous cell carcinoma, adenocarcinoma, SCLC, and large-cell carcinoma. *P*-values are indicated, calculated using the Fisher's exact test. (*C*) Prediction of overall survival probability in the human Affymetrix lung adenocarcinoma data set stratified for high (red lines) and low (black lines) RANK, RANKL, OPG, and LGR4 mRNA expression based on the best fit algorithm. Data were obtained using KM plotter. *P*-values (log rank test) are indicated. (*D*) RANK IHC in a patient-derived *KRAS* mutant adenocarcinoma used for xenograft experiments. Bars, 100 µm. (*E*) Growth of a patient-derived lung adenocarcinoma xenograft (PDX). PDX tumor fragments were implanted subcutaneously in the right flanks of 6- to 8-wk-old female NSG (NOD-*scid* IL2Rγ^null^) mice. At day 45 (when tumors had reached ∼200 mm^3^), mice were treated with 10 mg/kg OPG-Fc or PBS subcutaneously twice per week, and tumor volumes were measured twice per week using digital calipers. OPG-Fc treatment significantly reduced tumor burden. *P* = 0.024, repeated measures analysis of variance (RMANOVA) followed by Dunnett's post-hoc test. Data represent the mean ± SEM for each group. *n* = 10 per group. (*F*) Proliferation of PDX tumor cells in PBS and 10 mg/kg OPG-Fc-treated mice was determined using Ki67 immunostaining, analyzed at the termination of the experiment for ethical reasons. Data are shown as mean percentage of Ki67^+^ cells (±SEM) among tumor cells, comparing the two groups. *n* = 10 per group. *P* = 0.0143, unpaired *t*-test.

To test whether RANK could play a role in primary human lung cancer, we analyzed RANK expression in healthy lungs and primary lung cancer tissue using immunohistochemistry (IHC). In healthy human lung tissue (*n* = 10 individuals), RANK expression was not observed in any normal alveolar lung epithelium but expressed in larger bronchioles and alveolar macrophages. We next analyzed 120 human lung tissue samples of non-small-cell lung cancer (NSCLC) and small-cell lung cancer (SCLC) and detected strong tumoral RANK expression in each lung cancer histotype; the incidence and intensity of expression were greatest in adenocarcinomas, with 72% of lung adenocarcinomas staining positive for RANK (Fig. 1A; Supplemental Fig. S1A). We next isolated primary human EpCAM^+^ lung cancer cells using a disaggregation protocol to preserve RANK cell surface expression and function, demonstrating a positive correlation between the IHC analysis and RANK flow cytometry (*n* = 33) (Supplemental Fig. S1B,C). Expression data were confirmed in a second independent lung cancer cohort (Graz cohort, *n* = 60) (Supplemental Fig. S1D) and a third tumor tissue microarray panel of 354 individual patient samples from primarily early stage treatment-naive resected NSCLC (“Uppsala” cohort) (Botling et al. 2013), with both showing frequencies of RANK expression within lung cancer histotypes similar to that observed with the patient surveys in Supplemental Figure 1A.

The Uppsala cohort also allowed us to assess the correlation between RANK expression and *KRas* mutational status in the patients. In the Uppsala lung cancer cohort, RANK positivity was significantly associated with the presence of *KRas* mutations (Fig. 1B). No correlation was observed between RANK expression and smoking status (Fig. 1B) and the tumor stage (data not shown). Using human lung cancer databases (Gyorffy et al. 2013), we next queried for mRNA expression of RANK; expression of its ligand, RANKL; expression of OPG, a soluble molecular decoy for RANKL/RANK (Simonet et al. 1997); and expression of LGR4, a recently reported RANKL receptor that can counteract RANK activation and hence functions as a membrane-bound negative regulator (Luo et al. 2016). High RANKL, low OPG, and low LGR4 mRNA expression was indeed associated with worse prognosis (Fig. 1C). High RANK mRNAs also showed a tendency for poor prognosis, albeit this association did not reach statistical significance (Fig. 1C). Thus, in three independent human lung cancer patient cohorts, we observed frequent expression of RANK protein in all lung cancer histotypes, and RANK positivity correlated with *KRas* mutations. Moreover, in human lung cancer patients, gene expression profiles, indicative of an active RANK pathway, are associated with a poorer outcome.

### Pharmacologic RANKL/RANK blockade impairs human lung cancer growth

Since RANK expression correlated with the mutant *KRas* status, we tested whether pharmacologic blockade of RANKL/RANK would affect the in vivo growth of human *KRAS* mutant lung adenocarcinomas using one patient-derived xenograft (PDX) in immunocompromised *NSG* (NOD-*scid* IL2Rγ^null^) mice. The *KRas* mutational status (*KRas*^*G12C*^) was determined by exome sequencing. Since *NSG* mice lack lymphoid cells, any potential RANKL contribution from infiltrating adaptive immune components or antibody-dependent cell-mediated cytotoxicity (ADCC) can be excluded; of note, these mice still maintain myeloid cells. In the xenograft setting, RANKL inhibition using the molecular decoy OPG-Fc significantly inhibited tumor growth of a transplanted RANK-expressing *KRas* mutant lung adenocarcinoma, although these growth inhibitory effects were quantitatively small (Fig. 1D,E). Of note, we used OPG-Fc because it also blocks endogenous RANKL, whereas denosumab binds only to human RANKL. In line with reduced tumor growth, treatment of mice bearing RANK^+^ tumors with OPG-Fc also caused a significant reduction in the proliferation marker Ki67 within the tumor (Fig. 1F). The pharmacological in vivo efficacy of OPG-FC was demonstrated by a significant reduction in the osteoclast marker sTRAP5b (Supplemental Fig. S1E). These results indicate that RANK inhibition impairs the growth of human adenocarcinoma cells.

### RANK expression is induced in the murine lung epithelium by oncogenic KRas

**Figure 2.**
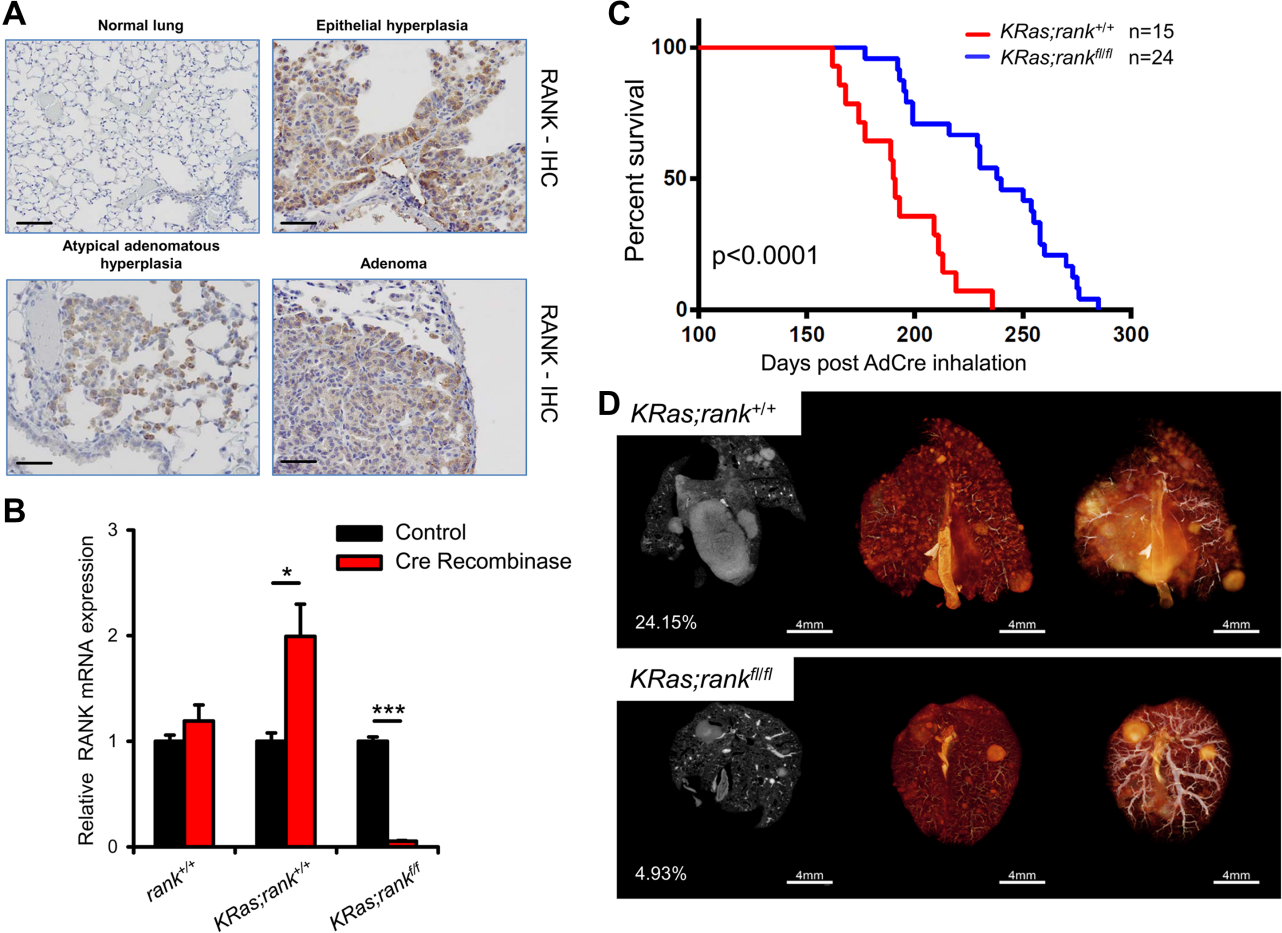
Loss of RANK prolongs survival of *KRas*^*G12D*^ -driven lung cancer. (*A*) Increased RANK expression during the progression of lung tumors. Analysis of RANK expression by IHC during lung tumor progression in *KRas*^*G12D*^ mice. Representative images for RANK expression in atypical adenomatous hyperplasia (AAH), epithelial hyperplasia (EH), and adenoma of AdenoCre (AdCre)-infected mice are shown. Normal lung parenchyma is shown for a mouse not infected with AdCre (normal lung). AAH and EH were from mice 11 wk after AdCre infection; adenomas were from mice 14.5 wk after infection. Bars, 100 µm. (*B*) Quantitative PCR analysis of RANK mRNA levels in primary pneumocytes purified from *KRas;rank*^+/+^ and *KRas;rank*^*f/f*^ mice; the experiment was performed with three technical replicates and repeated three times independently. Samples without AdCre infection in every genotype were set to 1, respectively. (*) *P* < 0.05; (***) *P* < 0.001, unpaired two-sided *t*-test. (*C*) Kaplan Meier survival curves for *KRas;rank*^+/+^ (*n* = 15; median survival 191 d) and *KRas;rank*^*fl/fl*^ (*n* = 24; median survival 239 d) littermate mice injected intranasally with AdCre (2.5 × 10^7^ plaque-forming units). *P* < 0.0001 (log rank test) between *KRas;rankl*^*fl/fl*^ and littermate controls. (*D*) Representative microCT images of lung tumors of *KRas;rank*^+/+^ and *KRas;rank*^*fl/fl*^ littermate mice assayed 25 wk after AdCre inhalation. Three different images of the same lung are shown, and the percentages of tumor to lung volumes are indicated. See also Supplemental Figure S3A.

To test whether RANK (encoded by the *Tnfrsf11a* gene) is also expressed in mouse lungs, we performed in situ RANK immunostaining. In the normal murine lung, RANK protein is expressed in bronchial epithelial cells and local immune cells, in particular in macrophages, but is absent in alveolar epithelial cells (Fig. 2A; Supplemental Fig. S2A). To test whether RANK might play a role in lung development, we crossed *rank*^*floxed/floxed*^ mice to SP-C Cre mice (Perl et al. 2009) to specifically delete *rank* in lung epithelial cells; both histological assessment of lung morphology and metabolic analysis of such mice using calorimetric cages indicated apparently normal lung structure and function after *rank* deletion (Supplemental Fig. S2B–D); the same was true for whole-body *rankl* and *rank* mutant mice.

We next analyzed RANK expression in a classic murine lung cancer model; namely, *Loxp-Stop-Loxp- KRas*^*G12D*^ mice (Johnson et al. 2001). Upon AdenoCre (AdCre)-mediated deletion and clonal induction of the mutant *KRas*^*G12D*^ allele, *Loxp-Stop-Loxp- KRas*^*G12D*^ mice develop NSCLC carcinomas in a stepwise process from epithelial hyperplasia and adenomas to adenocarcinomas (Jackson et al. 2001). Corresponding *KRas* mutations frequently occur in human lung cancer (Thomas et al. 2007). Importantly, in the murine *KRas*^*G12D*^- driven lung cancer model, we observed strong RANK expression during tumor progression, including RANK staining in preneoplastic adenomatous hyperplasias of both the alveoli and bronchioles in addition to uniform RANK expression in adenoma tumors (Fig. 2A). Moreover, induction of oncogenic *KRas* in cultured primary pneumocytes resulted in increased RANK expression; as a control, Cre transfection of wild-type primary pneumocytes without oncogenic *KRas* activation did not induce RANK expression (Fig. 2B). Thus, in mice, RANK expression is strongly induced in both oncogenic KRas-expressing pneumocytes in vitro and *KRas*-driven lung cancer cells in vivo, already observed at early stages of hyperplastic transformation.

### Inactivation of RANK impairs lung tumor progression in *KRas;rank*^*fl/fl*^ mice

**Figure 3.**
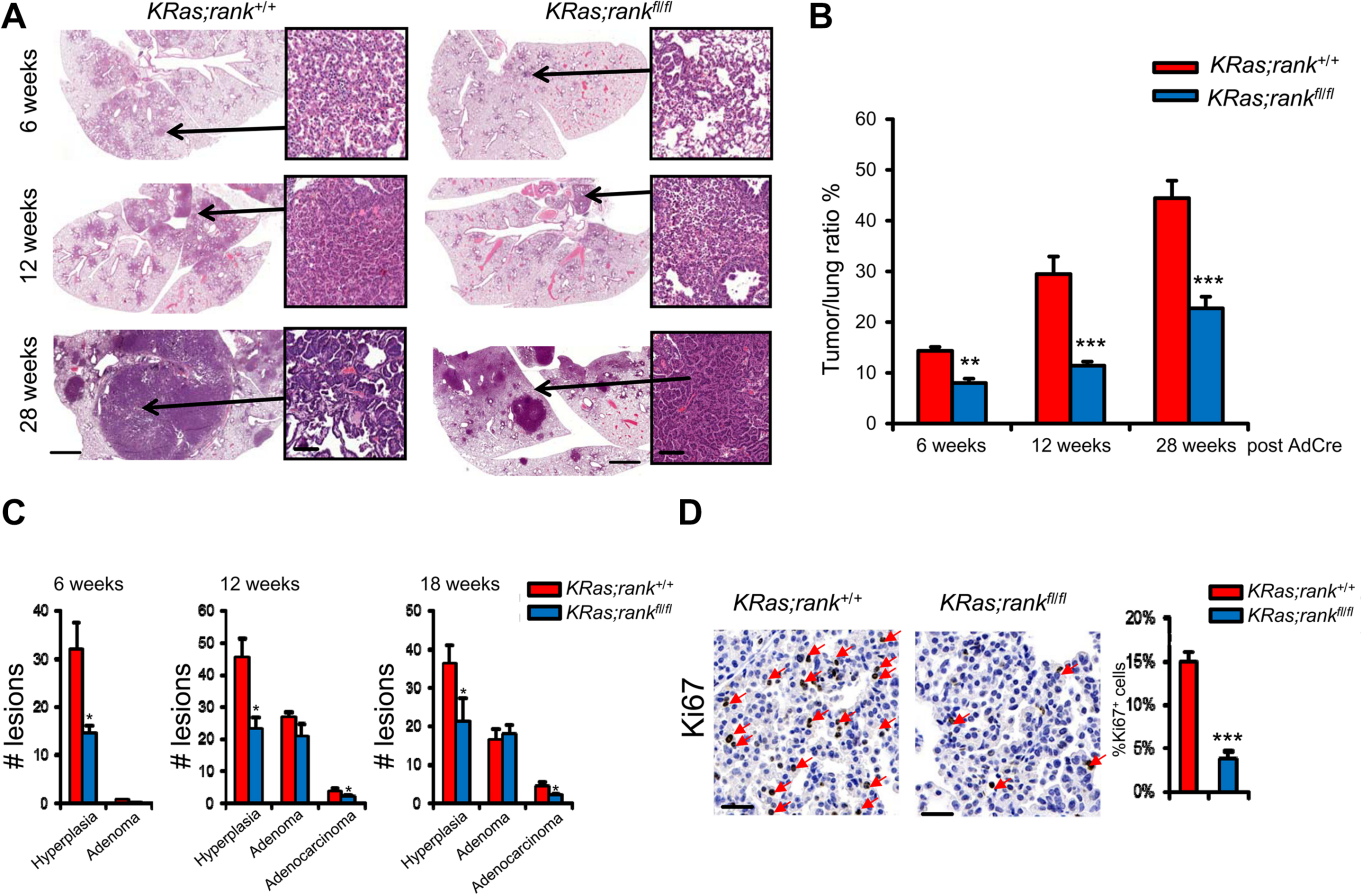
RANK controls lung tumor progression. (*A*) Representative histological lung sections and quantification of tumor to lung ratios of *KRas;rank*^+/+^ and *KRas;rank*^*fl/fl*^ littermate mice 6, 12, and 28 wk after AdCre inhalation. Images are shown for each genotype at the indicated time points. *Insets* in *A* show typical neoplastic foci. Bars: whole-lung section, 2 mm; *insets*, 50 µm. (*B*) At all three time points analyzed, *KRas;rankl*^*fl/fl*^ mice always exhibit significantly less tumor burden compared with their RANK-expressing littermate controls. *n* ≥ 8 mice per genotype at every indicated time point. (**) *P* < 0.01; (***) *P* < 0.001, χ^2^ test. (*C*) Quantification of hyperplasic regions, adenomas, and adenocarcinomas in *KRas;rank*^+/+^ and *KRas;rank*^*fl/fl*^ lungs at the indicated time points following AdCre instillation. *n* ≥ 6 for each cohort and time point analyzed*.* Data are shown as means ± SEM. (*) *P* < 0.05, χ^2^ test. (*D*) Representative images and quantification of Ki67^+^ tumor cells in *KRas;rank*^+/+^ and *KRas;rank*^*fl/fl*^ lung tumors analyzed 12 wk after AdCre infection. Quantification data are shown as mean ± SEM. *n* = 6. (***) *P* < 0.001, χ^2^ test.

To experimentally explore a direct cell-autonomous role of RANK in primary lung carcinogenesis, we crossed *rank*^*floxed/floxed*^ mice (Hanada et al. 2009) to *Loxp-Stop-Loxp- KRas*^*G12D*^ mice (Johnson et al. 2001) to generate *KRas;rank*^*fl/fl*^ mice. AdCre lung instillation into *KRas;rank*^*fl/fl*^ mice results in concurrent clonal deletion of *rank* in pneumocytes that express oncogenic *KRas*^*G12D*^. *rank* deletion was confirmed by in situ immunostaining (Supplemental Fig. S2E). Of note, AdCre can also induce deletion of floxed alleles in lung immune cells, such as macrophages that express RANK. Intriguingly, selective deletion of *rank* resulted in significantly prolonged survival (Fig. 2C). In line with prolonged survival, tumor burden in *KRas;rank*^*fl/fl*^ mice was also significantly reduced at 6, 12, and 28 wk after AdCre inhalation; reduced overall tumor burden was confirmed using microCT analyses of the total lung (Figs. 2D, 3A,B; Supplemental Fig. S3A). We also clonally deleted RANKL in the *Loxp-Stop-Loxp- KRas*^*G12D*^ model using *rankl*^*floxed/floxed*^ mice (Nakashima et al. 2011). RANKL is a membrane-bound as well as soluble cytokine produced by various cell types (Leibbrandt and Penninger 2008). Lung tumor-specific deletion of *rankl* had no apparent effect on lung cancer survival (Supplemental Fig. S3B,C), indicating that RANKL can be provided by cells other than the transformed epithelium to activate RANK.

**Figure 4.**
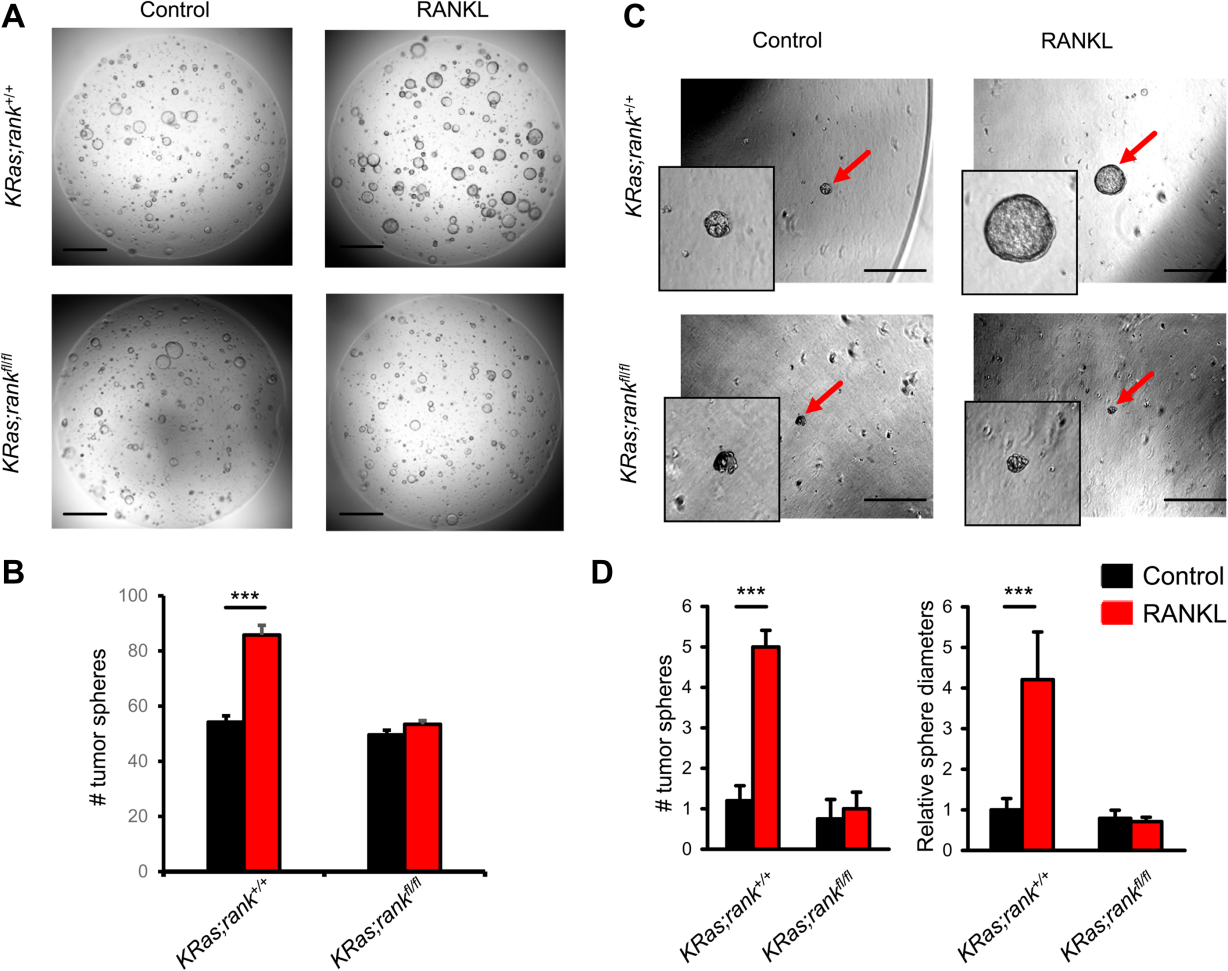
RANK drives lung cancer stem-like cell expansion. (*A*) Representative images of tumor spheroids derived from purified *KRas;rank*^+/+^ and *KRas;rank*^*fl/fl*^ primary lung tumor cells. Images were acquired 4 d after 1 µg/mL RANKL treatment. Five-thousand primary tumor cells were seeded per plate. The experiment was designed with six replicates for each condition and repeated with three different mice for each group, respectively. Bars, 1 mm. (*B*) Quantitative analysis (mean ± SEM) of tumor spheroid numbers described in *A*. (***) *P* < 0.001, unpaired two-sided *t*-test. (*C*) Representative images of tumor spheroids (red arrows) derived from purified *KRas;rank*^+/+^ and *KRas;rank*^*fl/fl*^ primary lung tumor cells. Images were acquired 7 d after 1 µg/mL RANKL treatment. Five-hundred primary tumor cells were seeded per plate. The experiment was designed with six replicates for each condition and repeated with three different mice for each group, respectively. Bars, 500 μm. (*D*) Quantitative analysis (mean ± SEM) of tumor spheroid numbers (*left* panel) and relative tumor spheroid diameters (*right* panel). The average diameters of tumor spheroids in *KRas;rank*^+/+^ without RANKL treatment was arbitrarily set as 1 to normalize all other groups. (***) *P* < 0.001, unpaired two-sided *t*-test.

We next assessed the initiation and malignant progression of lung tumors using established histopathological criteria (Jackson et al. 2001). At 6 wk after AdCre inhalation, *KRas;rank*^*fl/fl*^ mice exhibited significantly lower numbers of hyperplastic lesions than their littermates (Fig. 3C). At 12 and 18 wk after AdCre inhalation, we again observed significantly fewer hyperplastic regions as well as significantly reduced numbers of adenocarcinomas in *KRas;rank*^*fl/fl*^ mice (Fig. 3C). In line with enhanced tumor growth, proliferation, as detected by Ki67 staining, was markedly reduced in tumors from *KRas;rank*^*fl/fl*^ mice compared with their RANK-expressing littermates (Fig. 3D); however, there was no significant difference in the percentage of apoptotic cells between the two cohorts (data not shown). Comparative immunoprofiling of the early lung lesions from both *KRas;rank*^+/+^ and *KRas;rank*^*fl/fl*^ mice using flow cytometry 6 wk after AdCre inhalation also revealed no significant differences in the percentages of TCRαβ^+^ and TCRγδ^+^ T cells, NK cells, NKT cells, FoxP3^+^ regulatory T cells, B cells, IFNγ- and IL17A-producing inflammatory T cells, neutrophils, dendritic cells, and macrophages (Supplemental Fig. S4A–E). Since we already observed differences at the hyperplastic stage of cancer initiation (Fig. 3C), we assayed whether RANK might have a direct effect on the recently reported lung cancer stem-like cells using a three-dimensional (3D) organotypic tumor spheroid culture system (Tammela et al. 2017). Intriguingly, when we seeded primary lung tumor cells isolated from *KRas;rank*^+/+^ mice, RANKL stimulation markedly enhanced the numbers and sizes of lung cancer tumor spheroids, which did not occur in the absence of RANK expression (Fig. 4A–D). These data show that RANK can directly drive the expansion of *KRas*^*G12D*^ lung cancer stem-like cells and that deletion of *rank* results in impaired *KRas*^*G12D*^-driven cancer initiation and delayed malignant lung tumor progression.

### RANK affects mitochondrial bioenergetics in primary mouse pneumocytes and murine and human lung cancer cells

**Figure 5.**
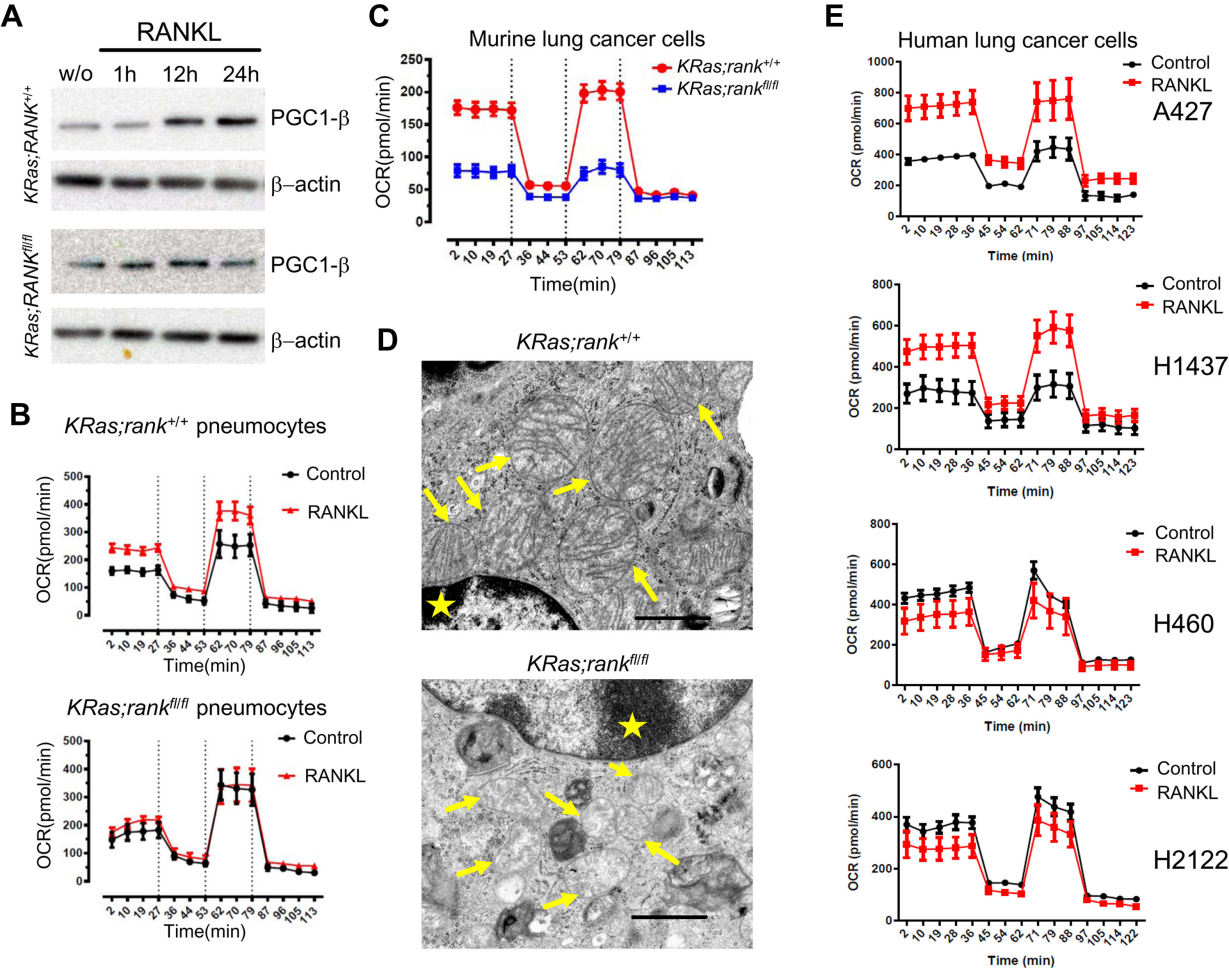
RANK couples to mitochondrial respiration. (*A*) Western blot analysis of pneumocytes isolated from *KRas;rank*^+/+^ and *KRas;rank*^*fl/fl*^ mice treated for 96 h with AdCre to induce *KRas* and delete *rank* to determine the induction of the PGC1β protein in response to RANKL stimulation at the indicated time points. (w/o) Without any treatment control. (*B*) Bioenergetics profiling of primary pneumocytes isolated from *KRas;rank*^+/+^ and *KRas;rank*^*fl/fl*^ mice. Purified pneumocytes were infected with AdCre to induce mutant *KRas* and delete *rank* in cells with *rank*^*fl/fl*^ alleles; these cells were then treated with 1 µg/mL RANKL ex vivo for 24 h and, as controls, left without RANKL treatment. Of note, we already observed induction of oxidative phosphorylation (OXPHOS) at RANKL doses as low as 100 ng/mL. Oxygen consumption rates (OCRs) ± SEM were recorded to construct bioenergetics profiles using Seahorse. A minimum of five replicates was analyzed for each condition, and three different mice for each genotype were used independently. (*C*) Bioenergetics profiling of purified primary lung tumor cells from *KRas;rank*^+/+^ and *KRas;rank*^*fl/fl*^ mice 18 wk after in vivo AdCre inhalation. Cells were treated with 1 µg/mL RANKL ex vivo for 24 h, and the assays used Seahorse. A minimum of replicates (OCR ± SEM) was analyzed for each condition, and purified primary lung tumor cells from three different mice were used independently. (*D*) Representative electron microscopy images for tumor tissues from *KRas;rank*^+/+^ and *KRas;rank*^*fl/fl*^ mice 28 wk after AdCre inhalation. Note the relatively normal mitochondria with mostly intact cristae in control tumors in contrast to swollen mitochondria with notable cristolysis in *rank* mutant lung cancer cells (arrows). Stars indicate nuclei. Bars, 5 μm. (*E*) Bioenergetics profiling of the indicated RANK^high^-expressing human lung cancer cell lines A427 and H1437 and the two RANK^low^ human lung cancer cell lines H460 and H2122 in response to 1 µg/mL RANKL. Cells were treated with RANKL for 2 h, and OCRs (±SEM) were recorded for at least six replicates for each condition using Seahorse. *X*-axes show the recording times in minutes. Experiments were repeated independently three times.

To explore the molecular mechanisms of how RANKL/RANK might affect the growth of lung cancer cells, we first isolated primary pneumocytes from *KRas;rank*^+/+^ and *KRas;rank*^*fl/fl*^ mice and infected them with AdCre to knock out RANK and at the same time induce oncogenic *KRas*^*G12D*^ (Supplemental Fig. S5A). These cells were then cultured in the presence of recombinant RANKL followed by RNA sequencing (RNA-seq). Gene expression profiling of *KRas;rank*^*fl/fl*^ and *KRas;rank*^+/+^ pneumocytes showed differences in their molecular signatures as assessed by gene clustering (all primary data were deposited to Gene Expression Omnibus [GEO] under accession no. GSE81670). Gene set enrichment analysis (GSEA) using C5 gene ontology (GO) and C2 reactome databases revealed that loss of RANK significantly up-regulated genes annotated primarily to tight junctions and down-regulated genes annotated to translation, DNA replication, and mitosis (Supplemental Fig. S5B; Supplemental Table S1). Intriguingly, numerous genes annotated to mitochondria structure and function, such as the TCA cycle and respiratory electron transport, were also markedly enriched by RANKL stimulation specifically in RANK-expressing pneumocytes (Supplemental Fig. S5C; Supplemental Table S1). We next assayed whether RANKL stimulation can induce expression of master regulators of mitochondrial biogenesis; namely, peroxisome proliferator-activated receptor γ coactivator 1-α (PGC1α) (Puigserver et al. 1998; Dorn et al. 2015) and PGC1β (Ishii et al. 2009; Dorn et al. 2015). We failed to detect any PGC1α induction in response to RANKL stimulation (data not shown). However, we observed a marked induction of PGC1β in *KRas;rank*^+/+^ primary pneumocytes upon RANKL stimulation, which was absent in *KRas;rank*^*fl/fl*^ cells (Fig. 5A). These data indicated that RANK can couple to mitochondrial homeostasis.

To test whether these observed changes indeed extend to altered mitochondrial respiration, we analyzed oxygen consumption rate (OCR) in primary pneumocytes (Gesta et al. 2011). In the presence of RANK, basal respiration, maximal respiratory capacity (an indicator of the level of oxidative phosphorylation [OXPHOS]), spare respiratory capacity (the Δ between basal and maximal capacity), and ATP production were markedly increased within 24 h after RANKL stimulation; none of these differences were detected in *rank* mutant pneumocytes (Fig. 5B; Supplemental Fig. S6A). Similar results were observed following 48 h of RANKL stimulation (data not shown). We next purified primary lung cancer cells from *KRas*;*rank*^+/+^ and *KRas;rank*^*fl/fl*^ mice 18 wk after AdCre inhalation and repeated these mitochondrial bioenergetics experiments. In line with our results in primary pneumocytes, we observed significant reductions in basal respiration, ATP production, maximal respiration, and spare respiration capacity in the absence of RANK expression (Fig. 5C; Supplemental Fig. S6B). Moreover, transmission electron microscopy of freshly isolated lung cancer cells revealed similar numbers of mitochondria but markedly altered mitochondrial structures with fewer cristae (Fig. 5D), indicating that mitochondria are both functionally and structurally impaired after genetic inactivation of *rank*.

To test whether our findings on the effects of RANK on OXPHOS extend to human lung cancer, we assayed mitochondrial bioenergetics in four human lung cancer cell lines; namely, in the two RANK^high^-expressing cell lines A427 and H1437 and the two RANK^low^-expressing cell lines H460 and H2122 (Supplemental Fig. S6C). Similarly to our data on murine primary pneumocytes and purified lung cancer cells, we observed a significant increase in mitochondrial respiration in A427 and H1437 cells upon RANK activation, whereas we did not find a significant change in the RANK^low^ H460 and H2122 human lung cancer cells (Fig. 5E; Supplemental Fig. S6D). Of note, RANK expression has been reported previously on other human lung cancer cell lines (Di Nunno et al. 2000; Chen et al. 2011). Thus, in primary nontransformed mouse pneumocytes and murine and human lung cancer cells, RANK can rewire mitochondrial bioenergetics.

### RANK drives lung cancer stem-like cell expansion via mitochondrial respiration

**Figure 6.**
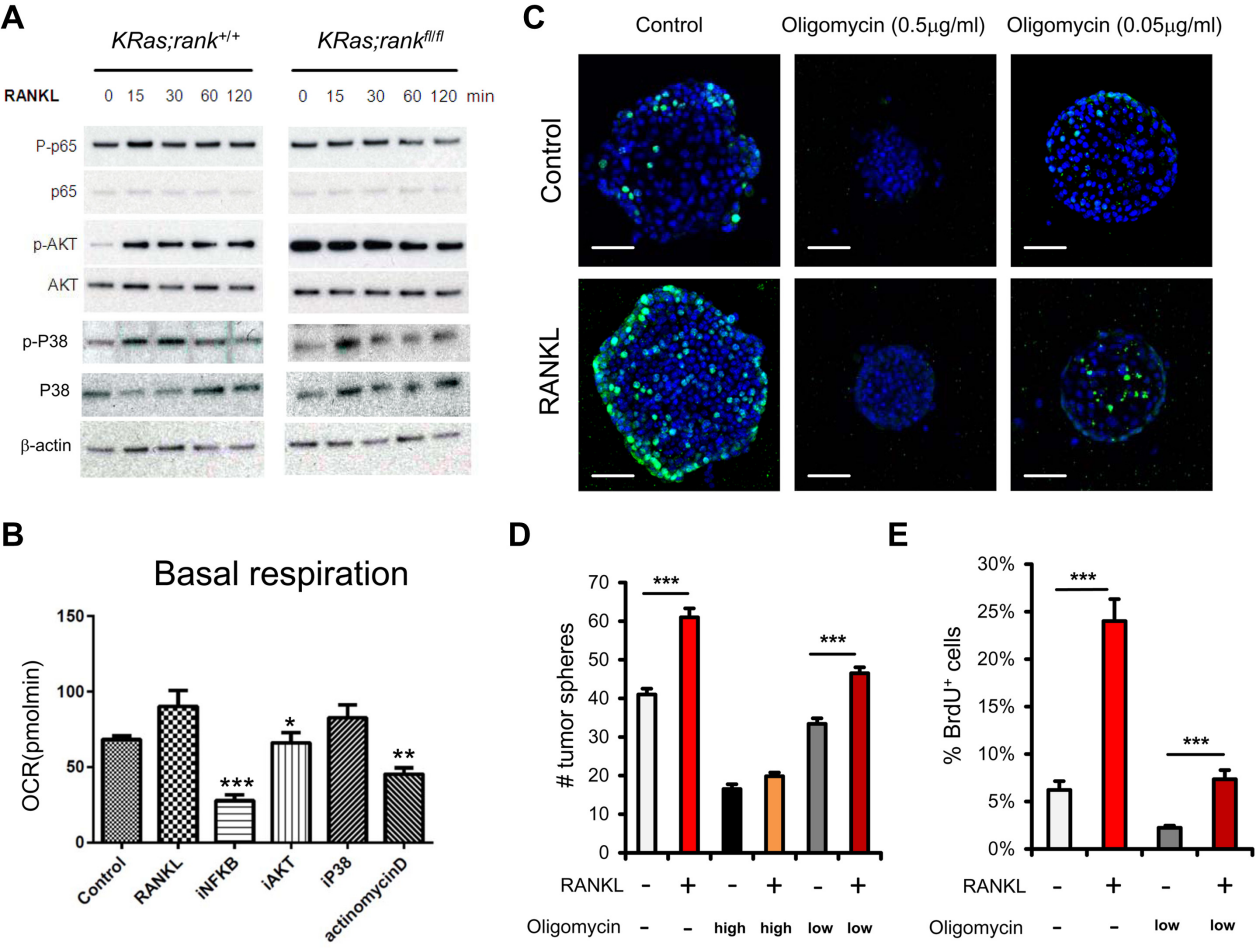
Mitochondrial respiration controls proliferation of lung cancer stem-like cells. (*A*) Western blotting of primary *KRas;rank*^+/+^ and *KRas;rank*^*fl/fl*^ pneumocytes to determine activation of the indicated signaling pathway in response to 1 µg/mL RANKL stimulation. Activation was determined at the indicated time points using phospho-specific antibodies. The respective total proteins are shown to control for protein expression. β-Actin is shown as a loading control. (*B*) Bioenergetics profiling of purified primary pneumocytes from *KRas;rank*^+/+^ mice. Mutant *KRas* was induced following AdCre infections, and cells were then left untreated (control) or treated with 1 µg/mL RANKL in the presence or absence of inhibitors to block NF-κB (iNF-κB), AKT (iAKT), P38 (iP38), and transcription (actinomycin D). In all experiments, cells were pretreated with the inhibitors. A minimum of five replicates was analyzed for each condition, and pneumocytes purified from two different mice were used independently. (*) *P* < 0.05; (**) *P* < 0.01; (***) *P* < 0.001, unpaired two-sided *t*-test. (*C*) Representative images for BrdU staining of tumor spheroids derived from *KRas;rank*^+/+^ primary lung tumor cells, which received no treatment or were treated with 1 µg/mL RANKL alone, oligomycin alone (low dose [0.05 µg/mL] or high dose [0.5 µg/mL]), and RANKL plus different oligomycin concentrations. Five-thousand primary tumor cells were seeded. BrdU labeling (10 µM/mL) was performed for 2 h. Experiments were performed with six replicates for each condition and repeated with three different *KRas;rank*^+/+^ mice. Sections were counterstained with DAPI. (*D*,*E*) Quantifications (mean ± SEM) of tumor spheroid numbers and BrdU^+^ cells within tumor spheroids from *C*. (***) *P* < 0.001, unpaired two-sided *t*-test. Bars, 50 µm.

How does RANK signaling couple to mitochondrial alterations? In osteoclasts, various signaling pathways have been identified essential for RANK-mediated osteoclast development (Feng 2005); however, in other cell types, different RANK activation cascades are engaged and relevant (Fata et al. 2000; Schramek and Penninger 2011). Therefore, we first tested what common RANK downstream pathways are induced in lung epithelial cells following RANKL stimulation. Biochemically, RANKL stimulation of *KRas*;*rank*^+/+^ primary pneumocytes showed induction of phospho-AKT, phospho-p65 NF-κB, and phospho-p38 (Fig. 6A). Stimulation of the RANK-expressing human tumor cell line A427 resulted in activation of the same downstream signaling pathways (Supplemental Fig. S7A), indicating that the same common activation cascades were engaged in murine and human lung epithelial cells. Other potential activation pathways remain to be determined. Moreover, we observed activation of p65 NF-κB, p38-MAPK, and AKT (as determined by phosphorylation using FACS) in freshly resected human lung tumor cells, correlating with the levels of RANK expression (data not shown).

Do these activation pathways play a role in the observed changes in mitochondrial respiration? Inhibitors of the NF-κB and AKT pathways, but not p38, abrogated the RANKL-induced increase in basal respiration in *KRas;rank*^+/+^ pneumocytes (Fig. 6B). These signaling pathways most likely converge on a RANKL/RANK-induced transcriptional response, consistent with our RNA-seq experiments, as inhibition of transcription by actinomycin D blocked the effects of RANKL on mitochondrial respiration (Fig. 6B). We then isolated lung cancer cells from KRas;*rank*^+/+^ and *KRas;rank*^*fl/fl*^ mice using RANKL induction of PGC1β as a readout (Supplemental Fig. S7B); inhibition of AKT and NF-κB, but not of p38, abrogated RANK-mediated PGC1β induction (Supplemental Fig. S7C). We next evaluated the effects of these inhibitors on lung cancer stem-like cell properties in 3D tumor spheroid assay. Again, we could detect enhanced tumor spheroids with RANKL stimulation; AKT inhibition almost completely blocked the spheroids’ formation, whereas P38 inhibition had no significant effect. NF-kB inhibition reduced the RANKL-triggered spheroid numbers to levels observed in the control cultures (Supplemental Fig. S7D,E). Finally, we tested whether this identified coupling of RANK to mitochondrial respiration had direct functional relevance for tumor stem-like properties using the 3D tumor spheroid assay. Interference with mitochondrial respiration using oligomycin at doses used previously in vitro and in vivo to block OXPHOS (Viale et al. 2014) indeed resulted in markedly reduced numbers of tumor spheroids in both control and RANKL-stimulated KRas;*rank*^+/+^ cells in a dose-dependent fashion (Fig. 6C,D). Moreover, whereas RANKL stimulation markedly enhanced proliferation in the tumor spheroids as determined by BrdU labeling, interference with OXPHOS significantly reduced the numbers of BrdU-positive cells (Fig. 6C,E). The effects of oligomycin on tumor sphere formation and BrdU labeling in *KRas;rank*^*fl/fl*^ cells are shown in Supplemental Figure S8. Thus, RANKL/RANK can induce lung cancer stem-like cell expansion via regulating mitochondrial respiration.

### Female sex hormones can affect *KRas*^*G12D*^-driven lung cancer via RANK

Epidemiologic studies have revealed differences between women and men, particularly in lung cancer etiology, progression, and treatment response, believed to be due to sex-related hormonal factors (Belani et al. 2007; Baik et al. 2011; Remon et al. 2014). Moreover, it has been reported that sex hormones can promote tumor progression in the *KRas*^*G12D*^;*p53*^*flox*^ mouse model (Hammoud et al. 2008). However, the molecular pathways for such effects were not known. Intriguingly, during the course of the human lung tumor data analysis, we observed that the association of better survival with low RANKL and high OPG expression was statistically more significant in female than in male cancer patients (Supplemental Fig. S9A). Of note, we did not observe such a difference for RANK expression, which is in line with the fact that expression of RANKL and OPG, but not RANK, are highly regulated by sex hormones. Moreover, in our IHC data set, we found significantly more tumoral RANKL expression in females with lung cancer (Supplemental Fig. S9B), supporting the notion that RANKL expression in lung cancer is biased toward women.

**Figure 7.**
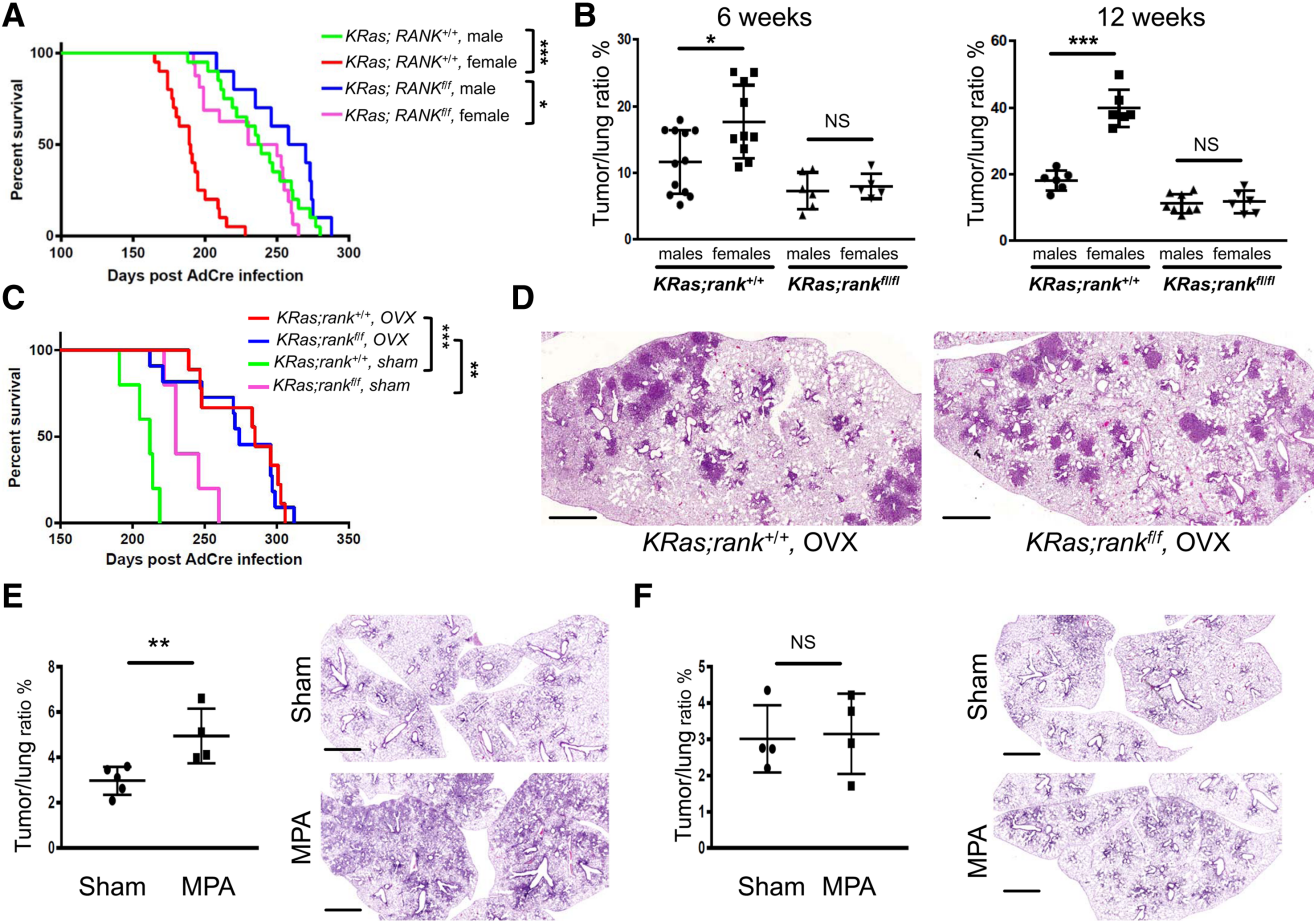
Female sex hormones can drive lung cancer via RANK. (*A*) Kaplan Meier survival curves for *KRas;rank*^+/+^ male (*n* = 20; median survival 238 d), *KRas;rank*^+/+^ female (*n* = 20; median survival 183 d), *KRas;rank*^*fl/fl*^ male (*n* = 10; median survival 262 d), and *KRas;rank*^*fl/fl*^ female (*n* = 16; median survival 215 d) mice injected intranasally with AdCre. (*)*P* < 0.05; (***) *P* < 0.001, log rank test. (*B*) Quantitative assessment of tumor to lung ratios among male and female *KRas;rank*^+/+^ and *KRas;rank*^*fl/fl*^ mice at 6 and 12 wk after AdCre inhalation. Data from individual mice are shown ±SEM. (*) *P* < 0.05; (***) *P* < 0.001, χ^2^ test. (*C*) Kaplan Meier survival curves for *KRas;rank*^+/+^ ovariectomized (OVX) (*n* = 9; median survival 285 d), *KRas;rank*^+/+^ sham-treated (*n* = 5; median survival 210 d), *KRas;rank*^*fl/fl*^ ovariectomized (*n* = 11; median survival 274 d), and *KRas;rank*^*fl/fl*^ sham-treated (*n* = 5; median survival 235 d) mice injected with AdCre. (**) *P* < 0.01; (***) *P* < 0.001 (between the ovariectomized groups and their respective *KRas;rank*^+/+^ and *KRas;rank*^*fl/fl*^ sham-treated littermate controls), log rank test. (*D*) Representative histological images of ovariectomized (OVX) *KRas;rank*^+/+^ and *KRas;rank*^*fl/fl*^ females 30 wk after AdCre inhalation. Bars, 2 mm. (*E*,*F*) Quantitative assessment of tumor to lung ratios and representative histological images of medroxyprogesterone acetate (MPA)-implanted (MPA) as well as sham-treated (Sham) *KRas;rank*^+/+^ (*E*) and *KRas;rank*^*fl/fl*^ (*F*) mice 8 wk after AdCre inhalation. MPA experiments were performed independently for the *KRas;rank*^+/+^ and *KRas;rank*^*fl/fl*^ cohorts. Of note, for the MPA experiments, we used a lower dose of the AdCre virus to better uncover possible MPA effects and assessed lung tumorigenesis at an early time point as a surrogate for tumor initiation. (**) *P* < 0.01, χ^2^ test. Bars, 2 mm.

We therefore analyzed survival curves in our lung cancer experiments (in the absence of *p53* mutations) according to gender. Indeed, we found that *KRas;rank*^+/+^ male mice survive significantly longer and have markedly less tumor burden than *KRas;rank*^+/+^ female mice at both 6 and 12 wk; in contrast, these survival differences were much less pronounced—with a comparable tumor burden at 6 and 12 wk—among *KRas;rank*^*fl/fl*^ female and *KRas;rank*^*fl/fl*^ littermate male mice (Fig. 7A,B). To test whether the differences in our female mice might depend on sex hormones, we removed female hormonal influence by ovariectomy (OVX) and then induced lung cancer via AdCre inhalation. After removing female sex hormones, the survival advantages of *KRas;rank*^*fl/fl*^ females disappeared completely (Fig. 7C), and histological analysis revealed no difference in tumor burden between the female *KRas;rank*^+/+^ and *KRas;rank*^*fl/fl*^ cohorts (Fig. 7D).

To finally assess whether female sex hormones can directly enhance the initiation of *KRas*^*G12D*^-driven lung cancer, we implanted RANK-sufficient female mice with the synthetic progesterone derivative (progestin) medroxyprogesterone acetate (MPA). MPA is commonly used for hormonal replacement therapy and contraceptives and has been demonstrated to strongly induce RANKL expression, thereby inducing the RANKL/RANK pathway (Schramek et al. 2010). Importantly, the Woman's Health Initiative trial study has shown that hormonal replacement therapy, especially the combination of estrogen with progestin, is associated with an increased number of deaths from lung cancer (Chlebowski et al. 2009). MPA significantly enhanced tumor burden in *KRas;rank*^+/+^ female mice (Fig. 7E). Most importantly, in the absence of RANK expression, MPA had no additional effect on lung tumor burden in *KRas;rank*^*fl/fl*^ littermate females (Fig. 7F). These data indicate that female sex hormones can directly affect *KRas*^*G12D*^-driven lung cancer, which appears to be regulated via RANK.

## Discussion

Lung cancer has become the most common neoplasm worldwide, with median and 5-yr survival expectations in advanced disease of ∼1 yr and <5% of patients, respectively (Siegel et al. 2015). Bone metastases are common in lung cancer. Therefore, RANK inhibition has been used in a phase III clinical trial to treat skeletal-related events in lung cancer patients (Scagliotti et al. 2012). Surprisingly, RANK inhibition with the monoclonal antibody denosumab, which binds to RANKL and therefore blocks binding of RANKL to RANK, resulted in significantly prolonged survival, especially in patients with NSCLC adenocarcinomas and squamous tumors. Importantly, the survival advantage of denosumab observed in the clinical study of lung cancer bone metastasis patients occurred in patients either with or without visceral metastasis, suggesting that a mechanism beyond the bone targeted effect of RANK blockade may be operative (Scagliotti et al. 2012). We show that clonal inactivation of *rank* in lung epithelial cells significantly affects lung cancer development, ultimately resulting in prolonged survival. RANK inhibition also reduces growth of human lung cancer in a PDX model. Thus, we uncovered a novel and unexpected function for RANK in primary lung cancer development. Genetically, it appears that RANKL does not need to be expressed in the lung cancer cells per se, indicating that systemic soluble RANKL and/or local RANKL production by cells in the tumor microenvironment provides this critical ligand.

Mechanistically, RANKL/RANK can rapidly alter mitochondrial respiration in primary mouse *KRas*^*G12D*^ mutant pneumocytes and in lung cancer cells, which at least in part could be explained by the marked induction of PGC1β expression. Moreover, RANK stimulation markedly enhances the formation of tumor spheroids from lung cancer progenitor cells, an effect that can be blocked by inhibition of mitochondrial respiration. Of note, it has been reported recently—albeit without describing the molecular pathway—that osteoclasts can change mitochondrial bioenergetics during their course of development (Nishikawa et al. 2015), although it was unclear whether this was a direct RANK activation effect or the consequence of the maturation of hematopoietic lineage-derived osteoclasts, and it was also not known whether such coupling might also occur in epithelial cells. Our data suggest that coupling of RANK to mitochondrial bioenergetics might constitute a common downstream pathway in different cell types. Whether PGC1β expression is also induced in osteoclasts needs to be determined. Moreover, we report here that RANK can trigger mitochondrial respiration in human lung cancer cell lines. Besides rewiring of mitochondrial energy homeostasis, RANK most likely drives lung cancer initiation and progression via other pathways, such as coupling to the cell cycle machinery, cell adhesion, or amplification of Wnt-responsive progenitor cells (Joshi et al. 2015).

Since RANKL/RANK/OPG are regulated via sex hormones, explaining, for instance, how progesterone can drive breast cancer (Schramek et al. 2010; Nolan et al. 2016; Sigl et al. 2016) and how sex hormones influence bone metabolism (Manolagas et al. 2014), we speculated that this system might also be involved in the long speculated and proposed gender differences in lung cancer (Chlebowski et al. 2009), lung cancer having become the number one “killer” for women with cancer (Siegel et al. 2015). However, to our knowledge, no molecular pathway has ever been reported to explain how gender-specific sex hormones might affect lung cancer development. We therefore speculated that RANKL/RANK might be involved in gender differences in lung cancer, although there was no factual evidence to support this notion. Intriguingly, when we analyzed male versus female mice, all animals being syngeneic littermates on the C57/Bl6 background, we observed gender-specific differences in lung cancer survival. Removal of endogenous sex hormones in female mice eliminated the survival advantages conferred by RANK deficiency, whereas, importantly, exposure to the synthetic progesterone MPA enhanced lung cancer initiation dependent on RANK expression. Sex hormone regulation of RANKL/RANK might in part explain the gender-specific differences observed in human lung cancer, thus identifying the first genetically verified pathway that shows how female sex hormones might drive lung cancer. Of note, we did not observe any apparent differences in RANK expression in the normal lungs or *KRas*^*G12D*^-driven lung tumors, and RANK expression could be induced in both male and female pneumocytes upon induction of oncogenic *KRas*. Moreover, the mitochondrial defects that we observed in the *rank* mutant lung tumors were present in both genders, and we observed apparently similar induction of PGC1β in male and female pneumocytes, although we cannot exclude subtle differences between the genders. Whether male sex hormones also affect lung cancer via RANKL/RANK, which are also regulated by testosterone (Hyde et al. 2012), needs to be determined. It would be also interesting to test in future experiments using different lung cancer models whether RANK might affect regional and cell type-specific differences in lung tumorigenesis.

We also report that RANK is frequently and highly expressed on primary human lung tumors and that a molecular signature of high RANKL and high RANK expression and low expression of OPG and LGR4, the natural endogenous inhibitors of the RANKL/RANK pathway, is associated with worse prognosis for lung cancer patients, in particular females, mirroring our genetic mouse data. Our human population data and experimental genetic mouse models suggest a scenario in which RANK is induced in both male and female lung epithelial cells by activating oncogenic *KRas* and in both male and female lung tumor cells coupled to mitochondrial respiration. RANKL and OPG levels are markedly regulated by female sex hormones, RANKL being regulated via progesterone, which we recapitulated in our MPA experiments. Thus, RANKL/OPG regulation by female sex hormones can create a milieu in which RANK is being hyperactivated by the presence of increased RANKL, explaining how female sex hormones could drive lung tumorigenesis. Since a drug that blocks RANKL has already been approved for human use with a very good safety profile, our findings are of direct clinical relevance and offer a feasible targeted approach for the treatment of primary lung cancer.

## Materials and methods

### Generation of *LSL-KRas*^*G12D*^;*rank*^*fl/fl*^ and *LSL-KRas*^*G12D*^;*rankl*^*fl/fl*^ mice

*rank*^*fl/fl*^ mice (Hanada et al. 2009) were generated at Institute of Molecular Biotechnology (IMBA), Vienna, and *rankl*^*fl/fl*^ mice were generated by Nakashima et al. (2011). These mice were backcrossed for at least 10 generations onto a C57Bl/6 background and then crossed to *LSL-KRas*^*G12D*^ mice (Johnson et al. 2001) to generate *LSL-KRas*^*G12D*^;*rank*^*fl/fl*^ and *LSL-KRas*^*G12D*^; *rankl*^*fl/fl*^ mice. In all experiments, only littermate mice were used as controls. All mice were maintained according to the ethical animal license protocol, complying with Austrian and European legislation. All experiments were approved by the Bundesministerium fuer Wissenschaft und Forschung, Austria (BMWF-66.015/0013-II/3b/2012).

### Induction of lung cancer

Inhalation of AdCre viruses was performed in 6- to 8-wk-old mice as reported previously (Schramek et al. 2011; Rao et al. 2014). For OVX studies, ovaries of 6- to 8-wk-old female mice were surgically removed, and, as controls, sham surgeries were performed. After 1 wk of recovery, mice received AdCre as reported previously (Schramek et al. 2011; Rao et al. 2014). For treatment with synthetic progesterone, 6- to 8-wk-old female mice were subcutaneously implanted with slow-release MPA pellets (50 mg per pellet; Innovative Research of America, NP161) as described previously (Schramek et al. 2010). One week later, mice were anesthetized and intranasally instilled with 5 × 10^6^ plaque-forming units of AdCre.

### Histology, IHC, and immunofluorescence staining

Histological analysis of lung tumors was performed as described (Schramek et al. 2011; Rao et al. 2014). RANK immunofluorescence staining was performed as described previously (Hanada et al. 2009).

### RANK and RANKL expression in human lung cancer

IHC was conducted in lung tumors, healthy tissue adjacent to the tumor, and tumor-infiltrating cells using specific monoclonal antibodies against human RANK (N-1H8 and N-2B10; Amgen, Inc.) and human RANKL (M366; Amgen, Inc.). IHC staining in the carcinoma element was quantified using H-scores (range 0–300), which incorporated staining intensity (range 0–3) and the percentage of positively stained tumor cells (range 0%–100%). Samples were defined as positive if the H-score was >0. Methods for RANK and RANKL IHC were essentially as described (Branstetter et al. 2012).

### Mitochondrial bioenergetics

Primary pneumocytes were prepared from 8-wk-old mice, and primary lung tumor cells were purified 18 wk after AdCre infection as described previously (Schramek et al. 2011). For primary pneumocytes, cells were infected with AdCre (multiplicity of infection [MOI] = 100) in vitro 4 d before Seahorse analysis. On the day of the experiment cells, were washed and incubated in 675 µL of XF assay medium (8.3 g/L DMEM base, 3.7 g/L NaCl, phenol red, 25 mM glucose, 2 mM L-glutamine at pH 7.4) for 1 h at 37°C. During the experiment, the oxygen concentration was measured every 6 min for a period of 2 min each. OCR was calculated using the Fixed Delta technique for determining the slope. The first three cycles in the experiment were used to determine basal mitochondrial respiration rates. After recording basal respiration, successive injections of 0.4 µg/mL oligomycin, 0.4 µM FCCP (carbonyl cyanide-p-trifluoromethoxyphenylhydrazone), and 0.6 µM rotenone, each followed by three cycles determining OCRs, were carried out to determine the basal mitochondrial respiration, ATP turnover, and maximum mitochondrial respiratory capacity by calculating the region under the curve. For inhibition experiments in primary pneumocytes, cells were treated with drugs to block NF-kB as follows: 10 µM QNZ ( EVP4593; Selleck, S4902), 10 µM AKT (MK-2206 2HCl; Selleck, S1078), 100 µM p38-MAPK (SB203580; Selleck, S1076), and 10 µM JNK (SP600125; Selleck, S1460). Transcription was blocked using 100 µM actinomycin D (ThermoFisher, 11805017). For all experiments, inhibitors were added 1 h prior to Seahorse analysis. For Seahorse experiments of human lung cancer cell lines, we used the following lines: The A427 human lung cancer cell line carrying a *KRasG12D* mutation and wild type for *p53*, H1437 cells that were *KRas* wild type but *p53* mutant, H460 cells carrying a *KRasQ61H* mutation and wild type for *p53*, and H2122 lung tumor cells carrying a *KRasG12C* mutation and mutant for *p53*.

### Western blotting

Western blotting was performed following standard protocols. Primary antibodies reactive to phospho-NF-kB p65 (1:1000; Cell Signaling, 3033), NF-kB p65 (1:1000; Cell Signaling, 4764), phospho-AKT (1:500; Cell Signaling, 4060), AKT (1:1000; Cell Signaling, 4685), phospho-p38 MAPK (1:800; Cell Signaling, 4631), p38 MAPK (1:1000; Cell Signaling, 9212), PGC1β (1:1000; Abcam, 176328), and β-actin (1:20,000; Sigma, F3022) were used. Primary antibody binding was detected by enhanced chemoluminescence (GE Healthcare, RPN2106).

### PDXs

All studies used 4- to 6-wk-old female NSG mice (Jackson Laboratories). The mice were housed five per filter-capped cage in sterile housing in an environmentally controlled room (temperature 23°C ± 2°C, relative humidity 50% ± 20%) on a 12-h light/dark cycle. The mice were fed commercial rodent chow (Harlan Laboratories, 2920X) and received filter-purified tap water ad libitum. Mice were individually identified by microchips (Bio Medic Data Systems) that were implanted subcutaneously at least 2 d prior to the study. PDX tumor fragments were implanted subcutaneously into mice at Jackson Laboratories and shipped to Amgen. Treatment (10 mg/kg PBS or OPG-Fc twice weekly; *n* = 10 per group) began when tumors were established and had reached a volume of ∼200 mm^3^. Tumor dimensions were assessed twice weekly with a Pro-Max electronic digital caliper (Sylvac), and tumor volumes were calculated using the formula length × width × height and expressed as cubic millimeters. Data are shown as mean ± SEM.

### Isolation of primary pneumocytes and lung tumor cells

Primary pneumocytes and lung tumor cells were purified as described previously (Schramek et al. 2011). For primary pneumocytes, lungs were dissected from 8-wk-old mice, infiltrated with IMDM containing 600 U/mL collagenase IV (Worthington) and 200 U/mL DNase (Worthington) through the trachea, and incubated for 1 h at 37°C; for lung tumor cells, the infiltration was performed with IMDM containing 5000 U/mL dispase (BD) and 200 U/mL DNase (Worthington) followed by same incubation condition.

### Gene expression profiling

mRNA-seq was performed as described previously (Rao et al. 2014). Genes differentially expressed between *KRas;rank*^+/+^ and *KRas;rank*^*fl/fl*^ tumor cells were selected using a cutoff at a log_2_ fold change of >1 and a *P*-value of <0.05 (false discovery rate-adjusted for multiple testing) and analyzed for functional enrichment using Ingenuity Pathway Analysis (IPA) and GO. Complementary to the overrepresentation analysis of differentially expressed genes, we applied GSEA to identify GO gene sets (MSigDB version 3.0 c5). mRNA-seq results are deposited under GEO accession number GSE81670.

### 3D tumor spheroid cultures

A flat round drop of Matrigel (Corning) was seeded in cell culture plates followed by incubation for 5 min at 37°C. Primary lung tumor cells were mixed with the Matrigel and kept on ice until they were seeded onto the droplet of Matrigel in the plate in “a droplet on a droplet” fashion. The Matrigel plug was incubated for 30 min at 37°C and then covered with cell culture medium. Images were acquired and analyzed 7 d later. For BrdU staining, tumor spheroids were incubated with 10 µM/mL BrdU for 2 h at 37°C followed by 3.7% formaldehyde fixation for 15 min at room temperature. Spheres were then permeabilized with PBST for 20 min and treated with 1 M HCl and 2 M HCl followed by neutralization with a phosphate/citric acid buffer. Tumor spheroids were then stained with an anti-BrdU antibody (Abcam, ab6326) overnight at 4°C, visualized with a fluorescence secondary antibody, and counterstained with DAPI.

### Quantitative RT–PCR

The following primers were used: mouse RANK forward primer (5′-CTTGGACACCTGGAATGAAG-3′), mouse RANK reverse primer (5′-CAGCACTCGCAGTCTGAGTT-3′), human RANK forward primer (5′- GACAAATGCAGACCCTGGAC-3′), human RANK reverse primer (5′- AGCTGGCAGAGAAGAACTGC-3′), human β-actin (forward) (5′-GGCTGTATTCCCCTCCATCG-3′), human β-actin (reverse) (5′-CCAGTTGGTAACAATGCCATGT-3′), mouse Gapdh forward primer (5′-GTCGGTGTGAACGGATTTGG-3′), and mouse Gapdh reverse primer (5′-GACTCCACGACATACTCAGC-3′).

### Statistics

All values are presented as means ± SEM. For the Kaplan Meier survival analysis, a log rank test was performed. To improve the robustness of our conclusions, we assessed genotype-related regression parameters for a significant deviation from zero and compared the model with the genotype regressor with an intercept-only model using a χ^2^ test. Quantifications of histological experiments were analyzed by means of a generalized linear mixed effects model with logit link by testing for nonzero regression coefficients and using a χ^2^ test. For statistical analysis of in vivo PDX efficacy studies, RMANOVA followed by Dunnett's post-hoc test for multiple comparisons was used. Statistical analyses were performed using JMP software version 8 interfaced with SAS version 9.1 (SAS Institute, Inc.). Of note, we applied several tests to one data set to increase our confidence in the reported biological findings. *P* < 0.05 was accepted as statistically significant.

## Supplementary Material

Supplemental Material
