# Supplementary material for: RANK rewires energy homeostasis in lung cancer cells and drives primary lung cancer
Source: Genes Dev. 2017 Oct 15;31(20):2099–112. doi: 10.1101/gad.304162.117 (PMC5733500; doi:10.1101/gad.304162.117)
Supplement: Supplemental Material [file supp_31_20_2099__index.html]

RANK rewires energy homeostasis in lung cancer cells and drives primary lung cancer — Supplemental Material 

# RANK rewires energy homeostasis in lung cancer cells and drives primary lung cancer

## Supplemental Material

- Supplemental\_Table\_S1.xlsx
- Supplemental\_Figure\_and\_Legends.pdf
