# Supplementary material for: RANK rewires energy homeostasis in lung cancer cells and drives primary lung cancer
Source: Genes Dev. 2017 Oct 15;31(20):2099–112. doi: 10.1101/gad.304162.117 (PMC5733500; doi:10.1101/gad.304162.117)
Supplement: Supplemental Material [file supp_gad.304162.117_Supplemental_Figure_and_Legends.pdf]

Supplemental Figure S1. RANK expression on human lung cancer cells.

(A) Incidence and H-Scores of RANK expression determined from IHC in the indicated primary lung cancer subgroups. The overall expression was generated using the H-scoring method which is the sum of the products of percentage of cells multiplied the intensity (0-3). Incidence was scored as the percent of tumors that showed any positive IHC signal. **(B)** Representative flow cytometry to detect RANK cell surface expression on EpCAM H1299 cells that do not express RANK, $EpCAM^+$ PC3 cells as RANK expressing positive control cells, and an $EpCAM^+$ resected primary NSCLC adenocarcinoma. Isotype control stainings are shown in the left panels. **(C)** Positive correlation between RANK flow cytometry results and IHC analysis on a subset of disaggregated primary human lung tumors (n=33). To confirm RANK flow cytometry results and to better understand the distribution of RANK expression, IHC was performed using a validated internal RANK IHC assay. H-Scores ranged from 0 to 260. **(D)** Representative images of RANK expression in a primary dissected NSCLC adenocarcinoma and a squamous lung tumor, using samples from a second lung cancer cohort (Graz cohort). Scale bars, 50µm and in insets, 20µm. **(E)** Detection of TRAP levels in the serum of PBS or OPG-Fc [10mg/kg] treated PDX mice assessed at study termination. TRAP levels indicate osteoclast activity. Data for individual mice are shown. Statistical comparisons were performed using Chi-square test.

Supplemental Figure S2. Deletion of RANK has no apparent effect on lung structures or lung function.

(A) Immunofluorescence staining to detect RANK protein expression in normal lung tissue. Note RANK protein (green) is primarily expressed in bronchial epithelial cells. DAPI (blue) was used

to counterstain for nuclei. Scale bars, 100μm. **(B)** Representative histological images of lung tissues isolated from *SP-C Cre;KRas;rank*$^{+/+}$ *and SP-C Cre;KRas;rank*$^{f/f}$ mice. Scale bars, upper panels, 2mm; lower panels 500μm. **(C)** qPCR analysis of RANK mRNA levels in primary pneumocytes purified from *SP-C Cre; KRas;rank*$^{+/+}$ *and SP-C Cre;KRas;rank*$^{f/f}$ mice. ***p<0.001 (Unpaired, two-sided t-test). **(D)** Comparison of average O_2 consumption and CO_2 production between *SP-C Cre;KRas;rank*$^{+/+}$ *and SP-C Cre;KRas;rank*$^{f/f}$ mice using the PhenoMaster module for indirect gas calorimetry. No significant differences were detected in O_2 consumption and CO_2 production; as a consequence the respiratory exchange rate (RQ) was also comparable between *SP-C Cre;KRas;rank*$^{+/+}$ *and SP-C Cre;KRas;rank*$^{f/f}$ mice. **(E)** Immunofluorescence staining to detect RANK protein expression (green) in lung tumors from *KRas;rank*$^{+/+}$ and *KRas;rank*$^{fl/fl}$ littermate mice 28 weeks after AdCre injections. Asterix indicates RANK expression in *KRas;rank*$^{+/+}$ tumor tissue. Note RANK expression in the bronchial epithelium (arrow) adjacent to the RANK deficient lung tumor in the *KRas;rank*$^{fl/fl}$ mouse. DAPI (blue) was used to counterstain for nuclei. Scale bars, 100μm.

Supplemental Figure S3. Epithelial deletion of RANK but not RANKL controls *Kras*^{G12D}-driven lung tumorigenesis.

(A) Representative 3D microCT movies of lung tumors of *KRas;rank*$^{+/+}$ and *KRas;rank*$^{fl/fl}$ littermate mice assayed 25 weeks after AdCre inhalation. The movies have been separately deposited, please click the links. **(B)** Kaplan Meier survival curves for *KRas;rankl*$^{+/+}$ (n=26, median survival=205 days) and *KRas;rankl*$^{fl/fl}$ (n=17, median survival=231 days) littermate mice injected intranasally with AdCre (2.5 × 10^7 PFU). There was no statistically significant difference

between the two cohorts (log rank test). **(C)** Representative histological images of lung tumors in *KRas;rankl*$^{+/+}$ and *KRas;rank*$^{fl/fl}$ mice, 6 and 30 weeks after AdCre inhalation. Scale bars, 2mm.

Supplemental Figure S4. Deletion of RANK has no apparent effect on intratumoral immune cells populations.

Comparative immunoprofiling of the early lung lesions from both *KRas;rank*$^{+/+}$ and *KRas;rank*$^{fl/fl}$ mice using flow cytometry 6 weeks after Ad-Cre inhalation. No significant difference was observed in in the assessed populations of T cells and NK cells **(A)**, Foxp3^{+} regulatory T cells **(B)**, B220^{+} B cells **(C)**, inflammatory T cells **(D)** and different myeloid cell subsets **(E)**.

Supplemental Figure S5. Gene expression profiling of *KRas;rank*$^{+/+}$ and *KRas;rank*$^{fl/fl}$ pneumocytes.

(A) qPCR analysis to control for RANK mRNA expression in primary pneumocytes isolated from *KRas;rank*$^{+/+}$ and *KRas;rank*$^{fl/fl}$ mice. Purified pneumocytes were infected with AdCre to induce mutant KRas and to delete RANK in cells with *rank*$^{fl/fl}$ alleles. Relative expression levels (+/-SEM) are shown as compared to RANK expressing *KRas;rank*$^{+/+}$ control cells (values set to 1). (**B** and **C**) Gene sets enriched in primary *KRas;rank*$^{+/+}$ and *KRas;rank*$^{fl/fl}$ pneumocytes using GSEA (normalized enrichment score (NES). Left, GSEA enrichment plot of the enrichment score (ES; y-axis) reflecting the degree of the gene sets overrepresentation in *KRas;rank*$^{+/+}$ and *KRas;rank*$^{fl/fl}$ pneumocytes at the extreme left or right of the entire ranked list. Solid bars represent genes of the

gene set. Right, GSEA-derived heatmap illustrating gene expression profiles of the leading edge subset. (**B)** Examples of gene sets over-represented in *KRas;rank*$^{fl/fl}$ pneumocytes (cell adhesion) or overrepresented in control *KRas;rank*$^{+/+}$ pneumocytes (mitosis). (**C)** Examples of enrichment for mitochondrial structure and mitochondrial respiration genes in *KRas;rank*$^{+/+}$ as compared to *KRas;rank*$^{fl/fl}$ pneumocytes.

Supplemental Figure S6. RANKL/RANK couple to mitochondrial respiration in primary pneumocytes and murine and human lung cancer cells.

(A) ATP production, basal respiration, maximal respiration and spare respiratory capacity (mean values +/- SEM) based on bioenergetics Seahorse profiling of purified primary pneumocytes from *KRas;rank*$^{+/+}$ and *KRas;rank*$^{fl/fl}$ mice. Purified pneumocytes were infected with AdCre to induce mutant *KRas* and to delete RANK in cells with *rank*$^{fl/fl}$ alleles; these *KRas;rank*$^{+/+}$ and *KRas;rank*$^{fl/fl}$ cells were then treated with RANKL [1µg/ml] for 24 hours and as controls left without RANKL treatment. A minimum of 5 replicates were analyzed for each condition and pneumocytes purified from 3 different mice were used independently. *$p<0.05$, **$p<0.01$ (Unpaired, two-sided t-test). **(B)** ATP production, basal respiration, maximal respiration, and spare respiratory capacity (mean values +/- SEM) based on bioenergetics profiling of purified primary lung tumor cells from *KRas;rank*$^{+/+}$ and *KRas;rank*$^{fl/fl}$ mice 18 weeks after *in vivo* AdCre inhalation. A minimum of 5 replicates (OCR +/- SEM) were analyzed for each condition and purified primary lung tumor cells from 3 different mice were used independently. See also Extended Fig. 3d. ***$p<0.001$ (Unpaired, two-sided t-test). **(C)** qRT-PCR analysis to determine RANK mRNA expression in the indicated human lung cancer cell lines. Relative RANK mRNA

expression levels (+/- SEM, n = 3) are shown as compared to human peripheral blood mononuclear cells (value arbitrarily set at 1). **(D)** Bioenergetics OCR profiling of ATP production, basal respiration, maximal respiration and spare respiratory capacity based on bioenergetics Seahorse profiling of the indicated human lung cancer cell lines. Cells were stimulated with RANKL [1μg/ml] for 2 hours (red lines and bars) and as controls left without RANKL treatment (black lines and bars). Data are all shown as mean values +/- SEM. A minimum of 6 replicates were analyzed for each condition and lung cancer cell line; experiments were independently repeated 3 times. *$p<0.05$, **$p<0.01$ (Unpaired, two-sided t-test).

Supplemental Figure S7. RANKL/RANK triggers lung cancer stem-like cells proliferation and mitochondrial homeostasis.

(A) Western blotting of A427 human lung cancer cells to determine activation of the indicated signaling pathway in response to RANKL stimulation [1μg/ml]. Activation was determined at the indicated time points using phospho-specific Abs to detect p65 NF-κB, AKT and p38-MAPK. The respective total proteins are shown to control for protein expression. β-actin is shown as a loading control. **(B)** Western blot analysis of purified *KRas;rank*$^{+/+}$ and *KRas;rank*$^{fl/fl}$ tumor cells to determine activation of the PGC1β in response to RANKL (1μg/ml) stimulation, analysed at indicated time points. β-actin is shown as a loading control. **(C)** Determination of PGC1β protein expression in purified *KRas;rank*$^{+/+}$ and *KRas;rank*$^{fl/fl}$ tumor cells stimulated with RANKL or RANKL plus inhibitors of AKT, P38 and NF-κB, respectively. **(D)** 3D tumor spheroids assay of purified *KRas;rank*$^{+/+}$ tumor cells treated with RANKL or RANKL plus inhibitors of AKT, P38

and NF-κB, respectively. 5000 primary tumor cells were seeded. Experiments were performed with 6 replicates for each condition and repeated with 3 different *KRas;rank*$^{+/+}$ mice. Scale bars, 1mm. **(E)** Quantification (mean values +/- SEM) of tumor spheroids numbers as shown in the representative images in **(D).** **p<0.01, ***p<0.001 (Unpaired, two-sided t-test).

Supplemental Figure S8. RANKL stimulation does not induce enhanced tumor sphere formation in *KRas;rank*$^{fl/fl}$ mutant tumor cells.

(A) 3D tumor spheroids assay of purified *KRas;rank*$^{fl/fl}$ tumor cells treated with RANKL [1μg/ml] or oligomycin alone [low dose is 0.05μg/ml, high dose is 0.5μg/ml] or RANKL plus different oligomycin concentrations. 5000 primary tumor cells were seeded. Experiments were performed with 6 replicates for each condition and repeated with 3 different *KRas;rank*$^{fl/fl}$ mice. Scale bars, 1mm. **(B)** Quantifications (mean +/- SEM) of tumor spheroids numbers of (**A)** N.S, not significant (Unpaired, two-sided t-test). **(C)** Representative images for BrdU staining of tumor spheroids derived from *KRas;rank*$^{fl/fl}$ primary lung tumor cells, which received no treatment, or were treated with RANKL alone [1μg/ml], oligomycin alone [low dose is 0.05μg/ml, high dose is 0.5μg/ml] and RANKL plus different oligomycin concentrations. 5000 primary tumor cells were seeded. BrdU labelling [10 μM/ml] was performed for 2 hours. Experiments were performed with 6 replicates for each condition and repeated with 3 different *KRas;rank*$^{fl/fl}$ mice. Sections were counter-stained with DAPI. **(D)** Quantifications (mean +/- SEM) BrdU^{+} cells within tumor spheroids as shown in **(C)**. N.S, not significant (Unpaired, two-sided t-test). Scale bars, 50μm.

Supplemental Figure S9. RANKL, RANK, and OPG expression in human lung cancer.

(A) Prediction of overall survival probability in males versus females in the human Affymetrix lung adenocarcinoma dataset stratified for high (red lines) and low (black lines) RANK, RANKL, and OPG mRNA expression based on the best fit algorithm for. Data presented were obtained using KM plotter. P values (log rank test) and total numbers of patients with either low (black lines) or high (red lines) RANK, RANKL and OPG expression, respectively, are indicated. **(B)** Cross-correlation matrixes to compare RANKL and RANK protein expression (determined by IHC) on human lung tumors with gender. n = 364, ”Uppsala” cohort with early stage treatment-naïve resected lung cancer, including SCC, AC, SCLC and LCC. P values are indicated, calculated using the Fisher’s Exact test.

Supplemental Table S1. Significantly enriched c5/GO gene sets (FDR <0.1) from a GSEA analysis of *KRas;rank*$^{fl/fl}$ versus *KRas;rank*$^{+/+}$ pneumocytes.

Rao_Supplemental Fig S1

A	Non-small Cell Lung Cancer								
	Adenocarcinoma			Squamous Cell Carcinoma			Small Cell Carcinoma		
	# of samples	% positive	Mean H-score	# of samples	% positive	Mean H-score	# of samples	% positive	Mean H-score
**RANK**	58	72	60	62	61	20	29	65	63

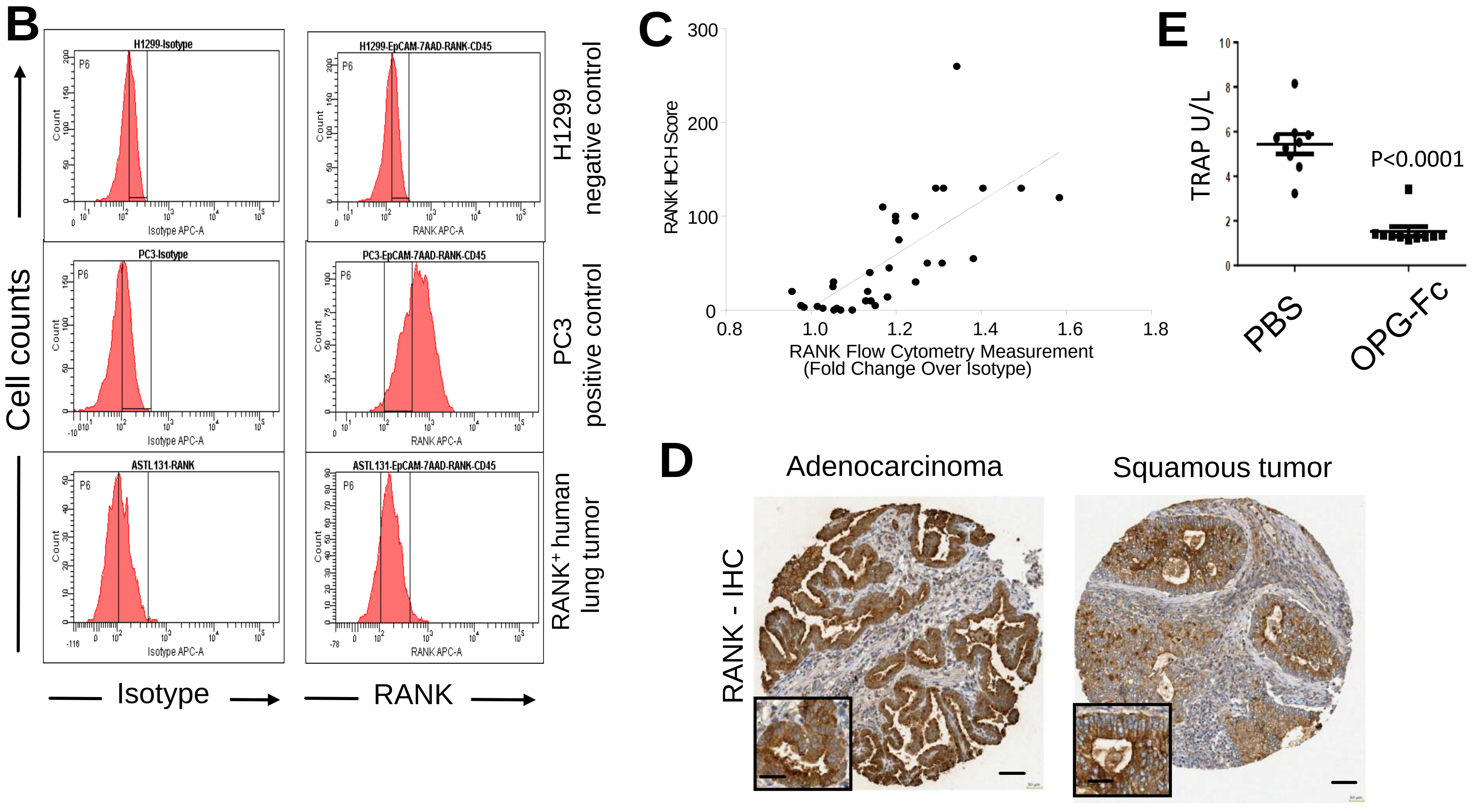

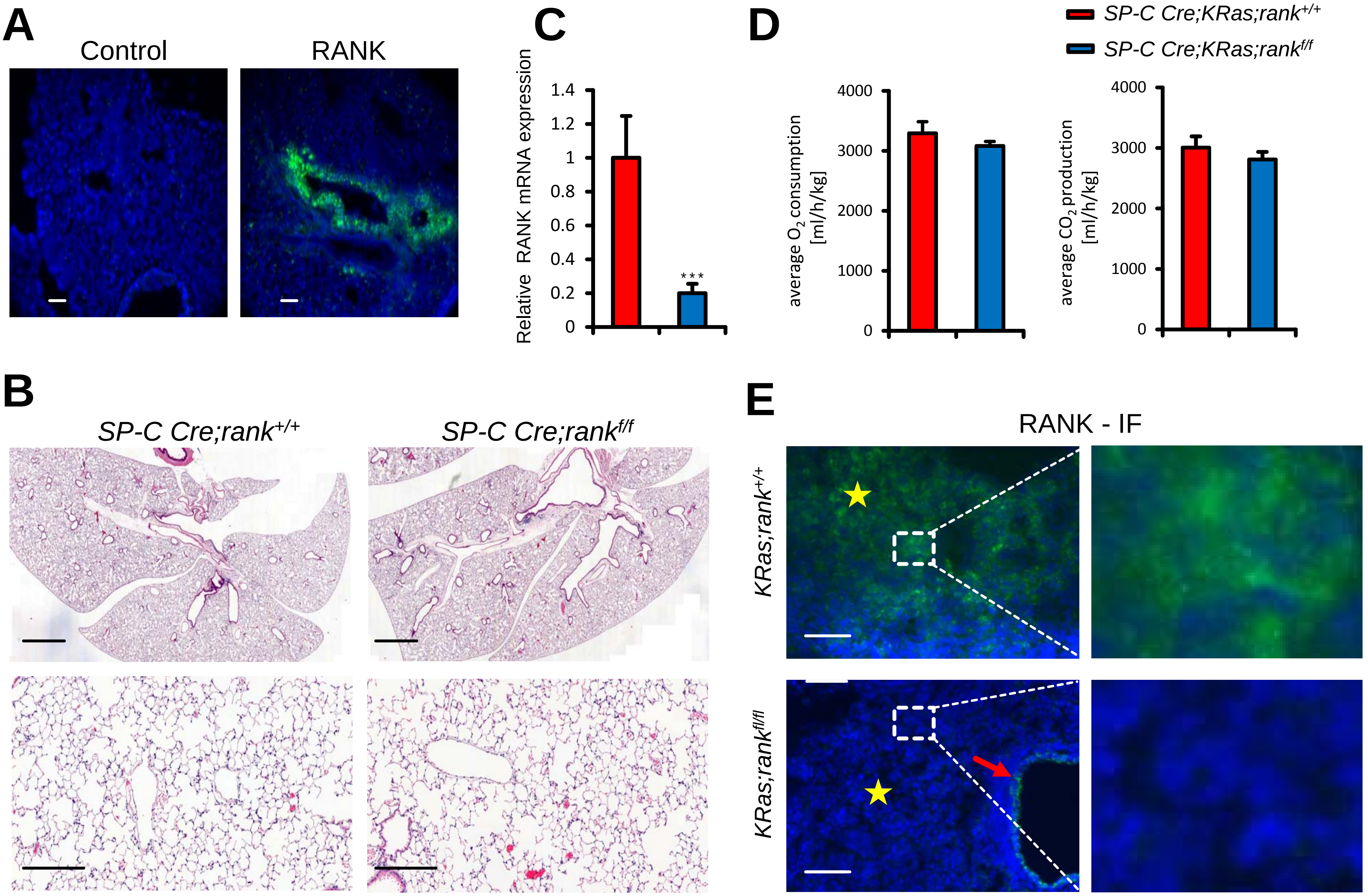
A
Control
RANK
B
SP-C Cre;rank+/+
SP-C Cre;rankf/f
C
Relative RANK mRNA expression
1.4
1.2
1
0.8
0.6
0.4
0.2
0
***
D
SP-C Cre;KRas;rank+/+
SP-C Cre;KRas;rankf/f
average O2 consumption [ml/h/kg]
average CO2 production [ml/h/kg]
4000
3000
2000
1000
0
E
RANK - IF
KRas;rank+/+
KRas;rankfl/fl

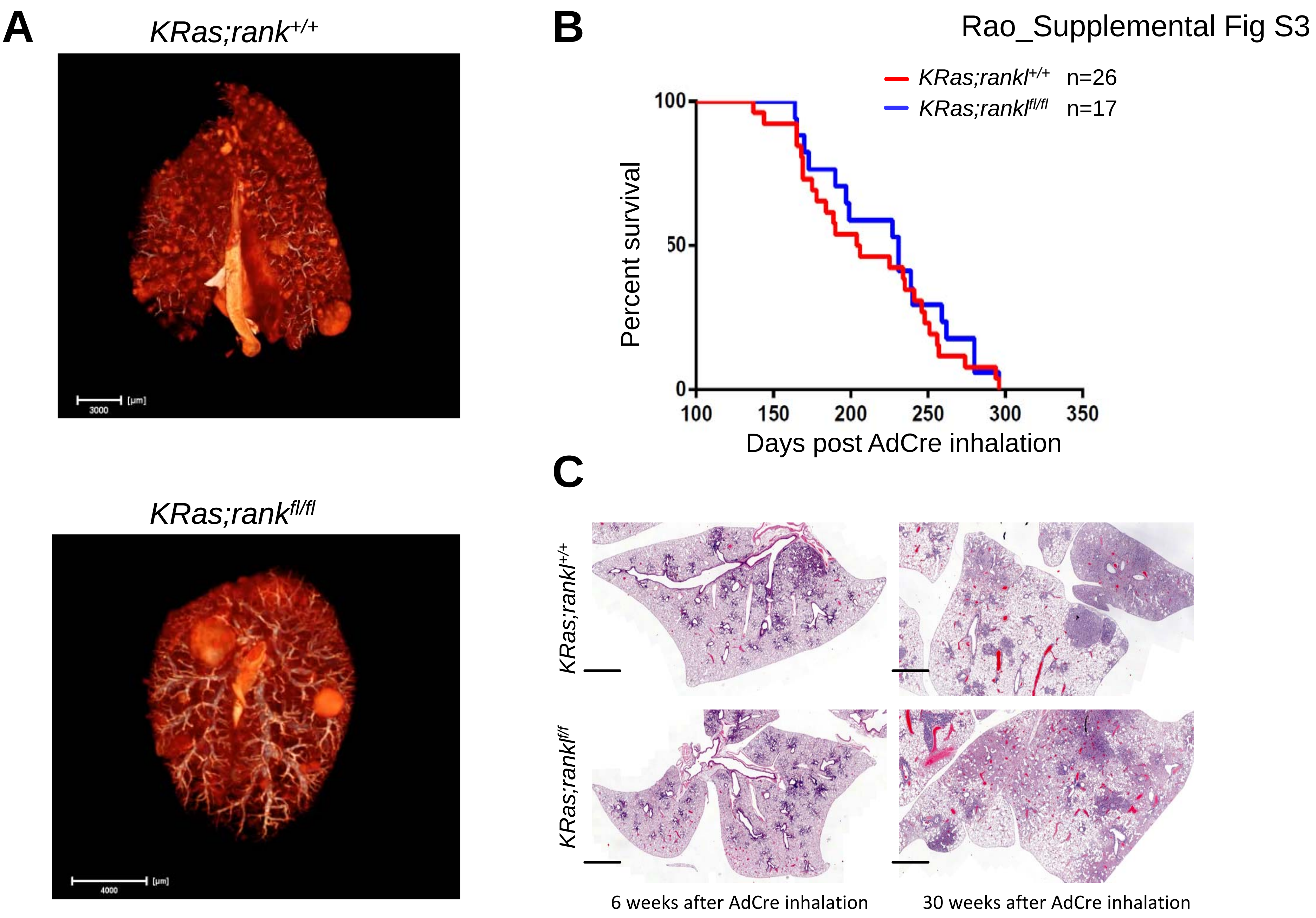
Rao_Supplemental Fig S3
A
KRas;rank+/+
3000 [μm]
KRas;rankfl/fl
4000 [μm]
B
KRas;rankl+/+ n=26
KRas;ranklfl/fl n=17
Percent survival
100
50
0
100 150 200 250 300 350
Days post AdCre inhalation
C
KRas;rankl+/+
KRas;ranklf/f
6 weeks after AdCre inhalation
30 weeks after AdCre inhalation

Rao_Supplemental Fig S4

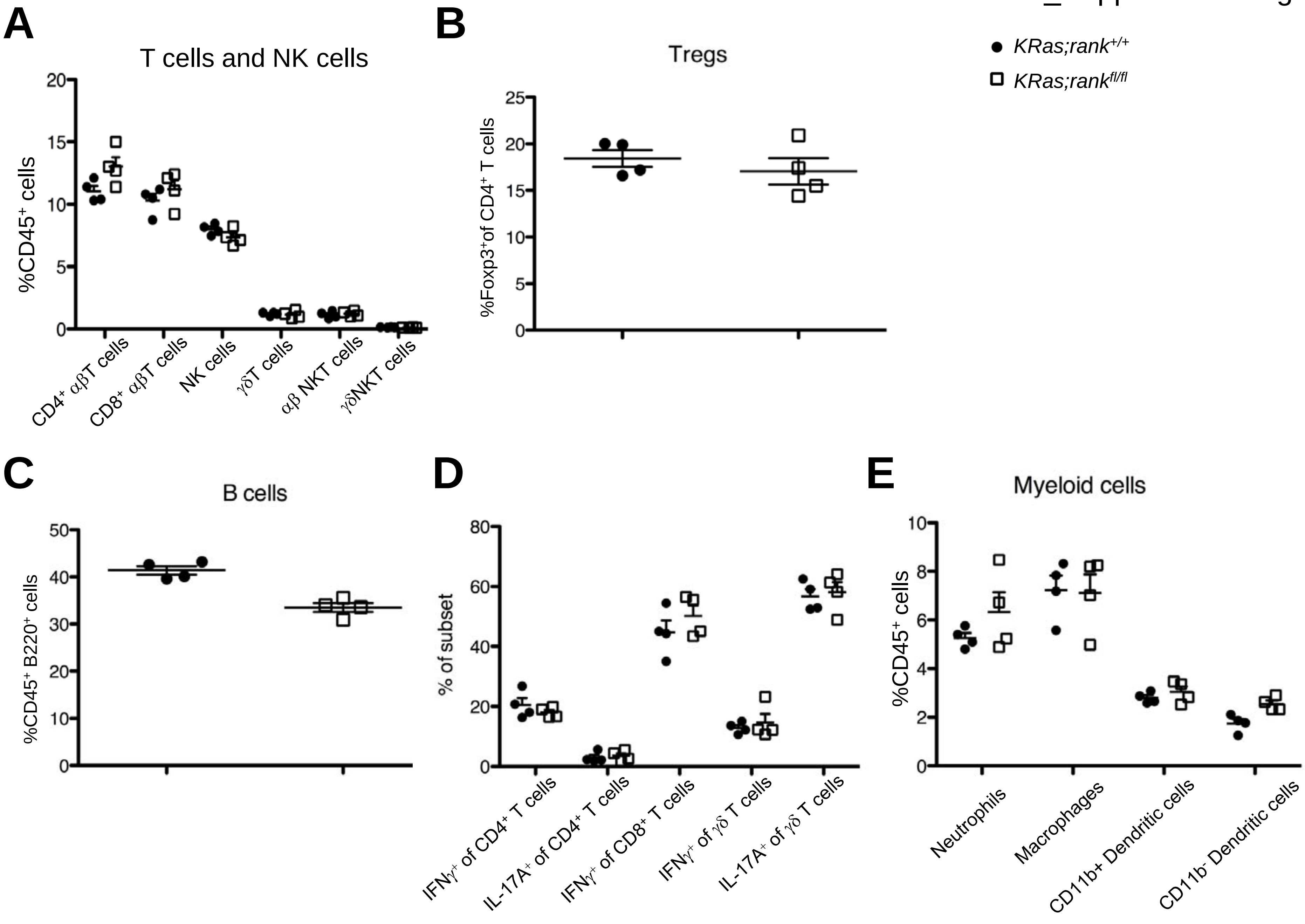

Rao_Supplemental Fig S5

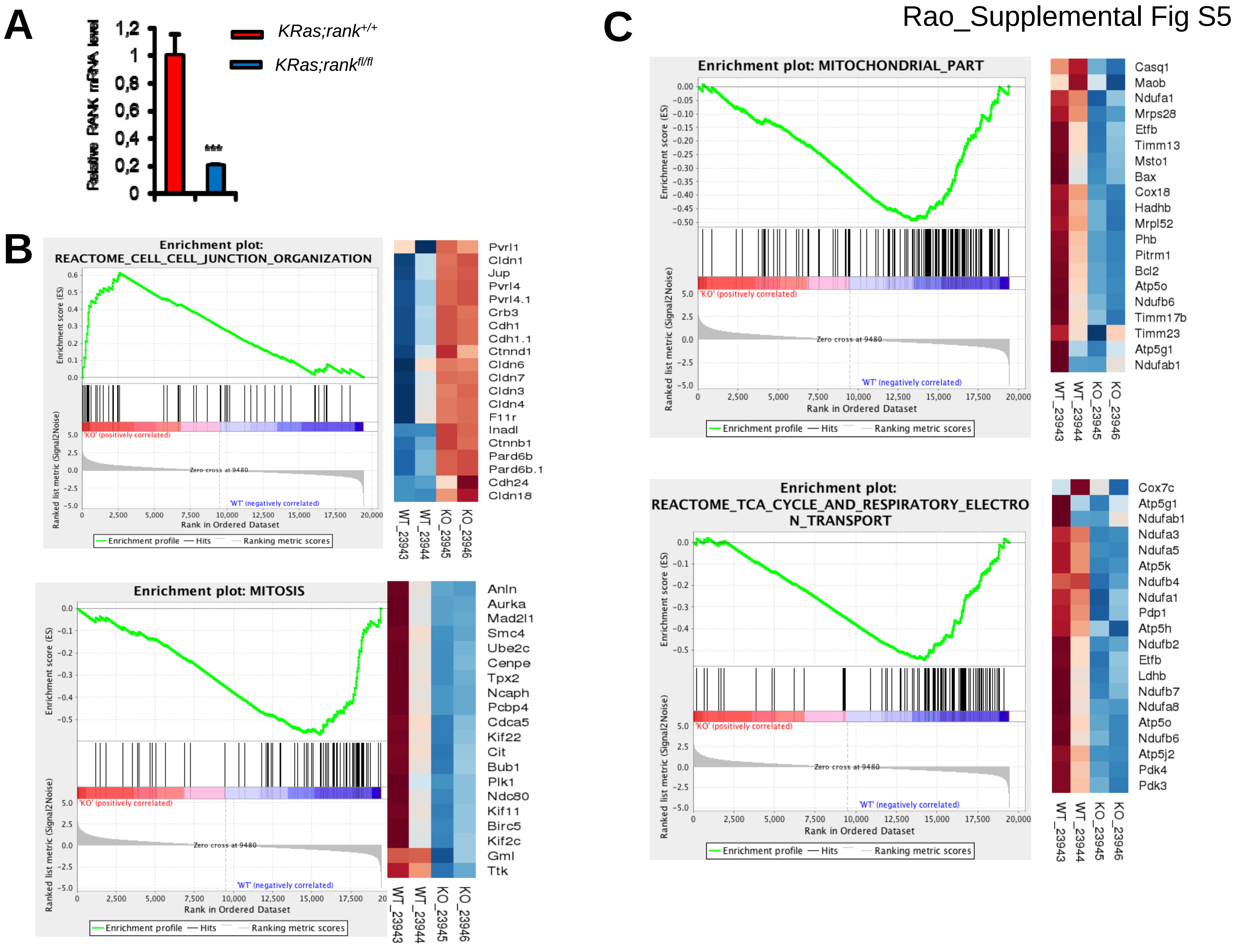

Rao_Supplemental Fig S6

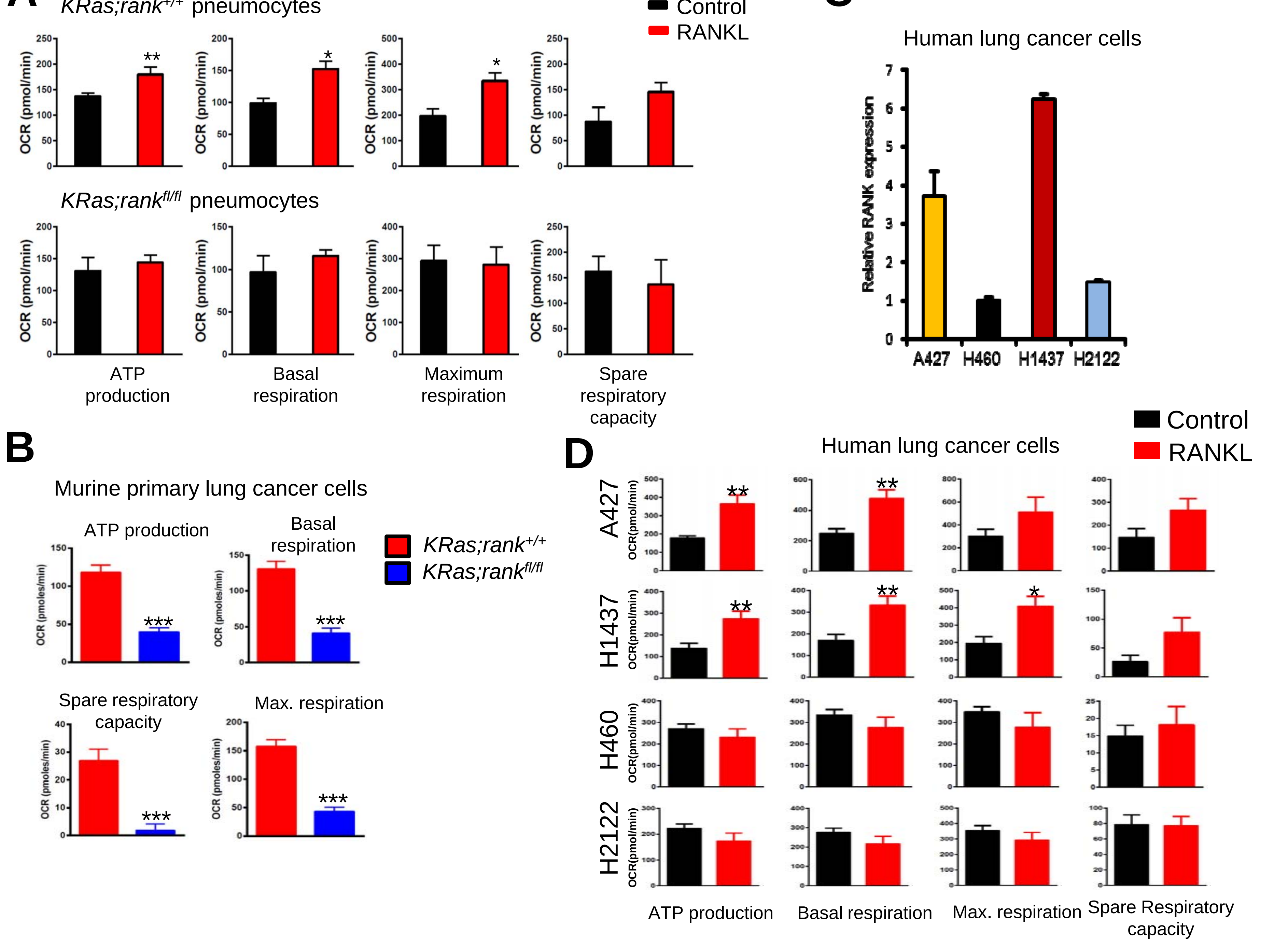

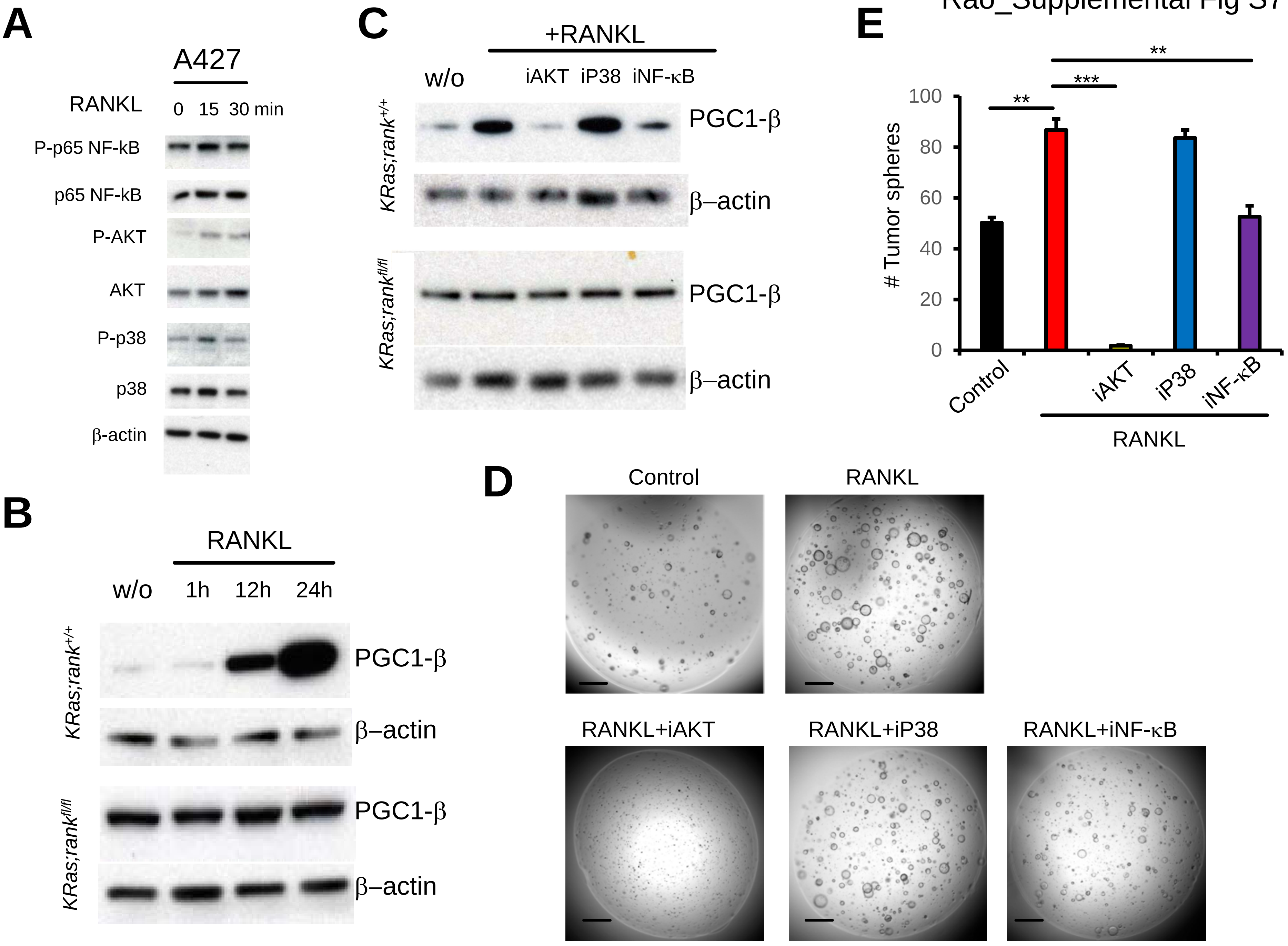
A
A427
RANKL
0 15 30 min
P-p65 NF-kB
p65 NF-kB
P-AKT
AKT
P-p38
p38
β-actin
B
RANKL
w/o
1h
12h
24h
KRas;rank+/+
KRas;rankfl/fl
PGC1-β
β–actin
C
+RANKL
w/o
iAKT
iP38
iNF-κB
D
Control
RANKL
RANKL+iAKT
RANKL+iP38
RANKL+iNF-κB
E
Tumor spheres
0
20
40
60
80
100
**
***
Control
iAKT
iP38
iNF-κB

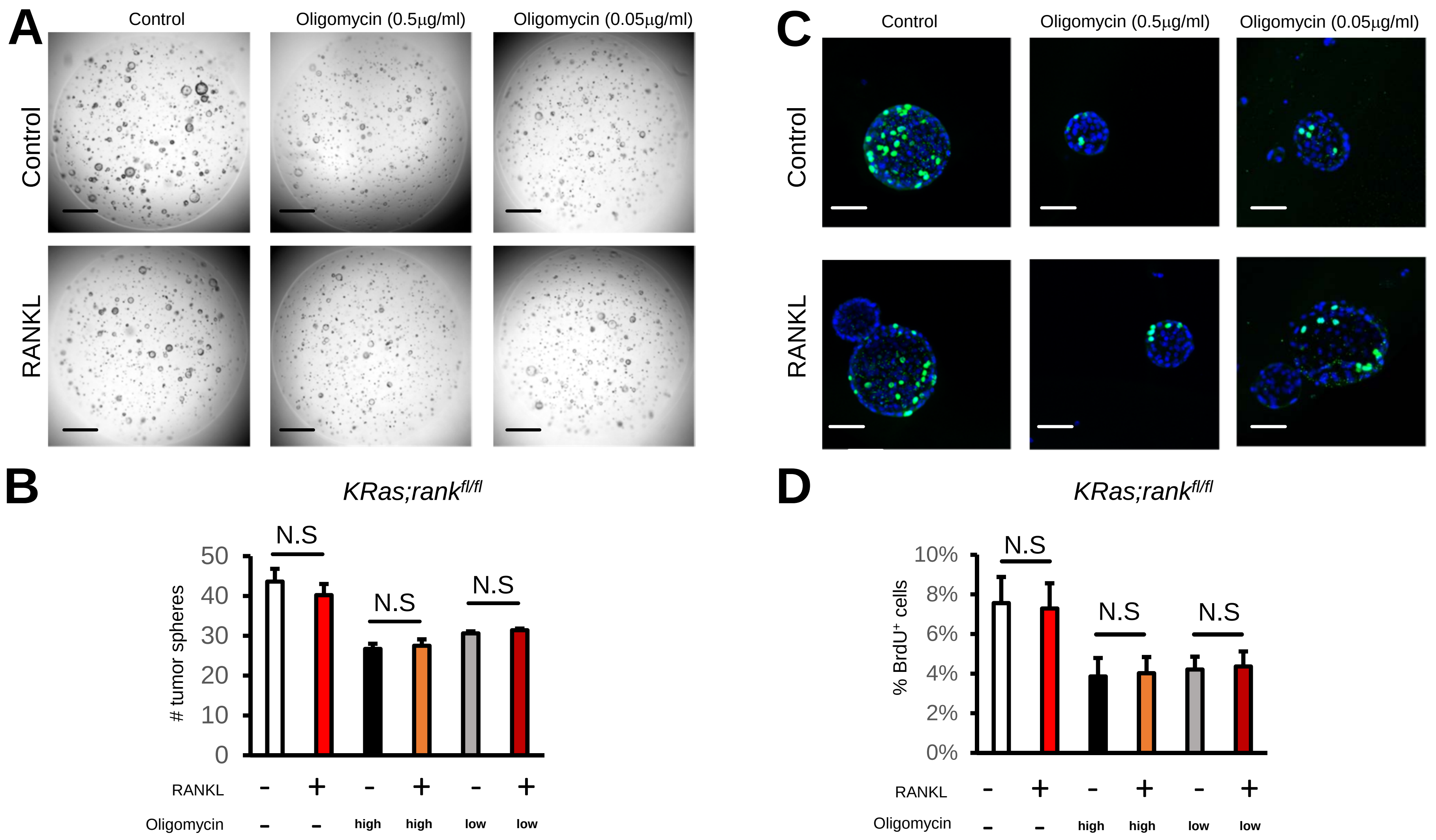

A
Control
Oligomycin (0.5μg/ml)
Oligomycin (0.05μg/ml)
Control
RANKL
B
KRas;rank^fl/fl
tumor spheres
50
40
30
20
10
0
N.S
N.S
N.S
RANKL
- + - + - +
Oligomycin
- - high high low low
C
Control
Oligomycin (0.5μg/ml)
Oligomycin (0.05μg/ml)
Control
RANKL
D
KRas;rank^fl/fl
% BrdU+ cells
10%
8%
6%
4%
2%
0%
N.S
N.S
N.S
RANKL
- + - + - +
Oligomycin
- - high high low low

Rao_Supplemental Fig S9

A

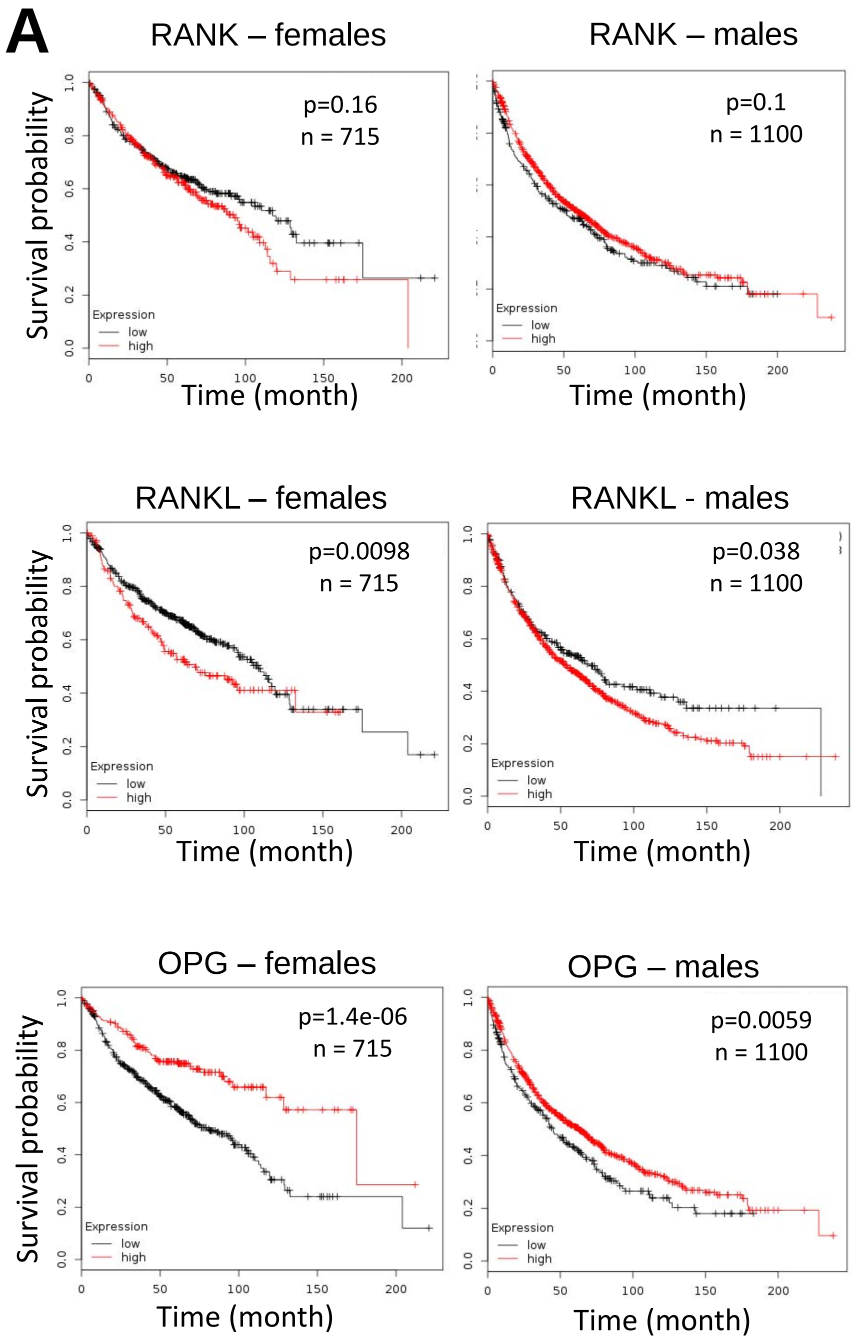

B

Gender

	Male	Female
RANK-	113 (60.4 %)	92 (56.1%)
RANK+	74 (39.6 %)	72 (43.9 %)

	Male	Female
RANKL-	87 (46.5 %)	55 (33.5 %)
RANKL+	100 (53.5 %)	109 (66.5 %)

Fisher´s exact test: p = 0.448 for RANK and gender and p = 0.01641 for RANKL and females.
